# AI-driven multimodal retinal imaging for early detection and risk stratification of vascular and neurodegenerative diseases

**DOI:** 10.1007/s00417-026-07273-6

**Published:** 2026-05-22

**Authors:** Lalit Agrawal, Pratik K. Agrawal, Shivam Sham Agrawal, Manoj Sheshrao Sonune, Rakesh K. Kadu, Madhusudan B. Kulkarni, Manish Bhaiyya

**Affiliations:** 1 Department of Computer Science and Engineering, Ramdeobaba University, Nagpur, Maharashtra 440013 India; 2 Symbiosis Institute of Technology, Nagpur Campus, Symbiosis International (Deemed University), Pune, Maharashtra 440008 India; 3 Electronics and Telecommunication Engineering, Ajeenkya DY Patil School of Engineering, Pune, Maharashtra 412105 India; 4 Manipal Institute of Technology, Manipal Academy of Higher Education, Manipal, India; 5 Department of Electronics and Telecommunication Engineering, Shri Sant Gajanan Maharaj College of Engineering, Shegaon, Maharashtra 444203 India

**Keywords:** Artificial intelligence, Retinal imaging, Multimodal learning, OCT, Cardiovascular disease, Neurodegenerative disease, Early detection, Risk stratification, Precision screening

## Abstract

Systemic vascular and neurodegenerative disorders are important causes of disability and death worldwide, mainly because of the late stage of diagnosis and the high cost of current screening tools. Artificial intelligence (AI) and multimodal retinal imaging offer a non-invasive and viable approach for early risk stratification and longitudinal monitoring. This review highlights how changes in the retinal vasculature and nerve layers are markers of underlying pathophysiologies related to cardiovascular, metabolic, and neurological disorders. It gives an account of the critical retinal imaging modalities, such as fundus photography, optical coherence tomography (OCT), OCT angiography (OCTA), and more recently developed metabolic-sensitive imaging modalities, and how current AI approaches, such as deep learning, self-supervised learning, and multimodal fusion, can be leveraged for better risk stratification and decision support. Evidence from hypertension, stroke, coronary artery disease, diabetic complications, Alzheimer’s disease, Parkinson’s disease, multiple sclerosis, and cognitive impairment shows the potential for the retina to serve as a scalable biomarker for systemic health. However, there are still hurdles to be cleared, such as multicenter validation, prospective clinical trials, data fusion, and regulatory frameworks. In conclusion, AI-assisted retinal analysis may make way for early screening, better prevention, and more accessible precision healthcare.

## Highlights

AI-enabled multimodal retinal imaging offers a non-invasive, scalable approach for early detection and monitoring of systemic vascular and neurodegenerative diseases.

Advanced techniques such as deep learning, self-supervised learning, and multimodal fusion significantly enhance risk stratification and clinical decision support.

Despite strong potential, challenges in validation, data integration, and regulatory frameworks must be addressed for real-world implementation.

## Key Messages

*****What is known***:**

- Retinal imaging (fundus, OCT, OCTA) reflects systemic vascular and neurodegenerative changes, enabling non-invasive disease assessment.

- AI techniques, particularly deep learning, have shown strong potential in analyzing retinal data for disease detection and risk prediction.

*****What is new***:**

- Integration of multimodal retinal imaging with advanced AI (self-supervised learning and multimodal fusion) enhances early risk stratification and longitudinal monitoring.
- Expands the role of retina as a scalable biomarker across diverse conditions including cardiovascular, metabolic, and neurodegenerative diseases.
- Highlights key translational gaps, including multicenter validation, data fusion challenges, and regulatory considerations, for real-world deployment.

## Introduction

Systemic vascular and neurodegenerative diseases remain the major, and inextricably linked, causes of global disability and healthcare expenditure. Cardiovascular diseases (CVDs) alone have an enormous mortality burden (e.g., ~ 19.8 million deaths in 2022, as estimated by the WHO), with a substantial number of these deaths attributable to heart attack and stroke, and a substantial number of these deaths occurring in low- and middle-income countries (who.int) [1–4]. Conversely, dementia is increasingly emerging as a concern owing to the aging population, with the WHO estimating ~ 57 million people living with dementia (2021) and nearly 10 million new cases every year, with Alzheimer’s disease accounting for the majority of cases. Metabolic disease further fuels these numbers, with the International Diabetes Federation estimating ~ 589 million adults living with diabetes in 2024, and rising. Outcomes for CVD, stroke, and neurodegenerative diseases remain inextricably linked to the time elapsed from the onset of risk factor detection and the initiation of interventions well before the development of any irreversible organ damage and loss of function (diabetesatlas.org) [5–7].

One of the biggest challenges is that the current diagnostic systems are prone to being delayed, costly, and not scalable for the proactive screening of the population on a large scale (Table 1) [8–12]. The current risk assessment for systemic vascular diseases would include the risk assessment based on the clinic-based evaluation of blood pressure, lipids, and glycaemic parameters, and further imaging studies based on clinical presentations [13–15]. The diagnosis of neurodegenerative diseases typically remains symptom-based and is aided by costly neuroimaging studies (MRI/PET) or by invasive biomarkers (such as cerebrospinal fluid analysis), making it difficult to implement early detection and longitudinal follow-up at a large scale [16]. This is especially true for resource-poor environments where access to specialists and advanced imaging is patchy, and a scalable, repeatable, and low-barrier screening method could help move the focus from late-stage detection to early intervention and prevention [17, 18].

**Table 1:** Evolution of screening/stratification approaches: conventional vs. modern (non-AI) vs. AI-enabled

Dimension	Conventional approach	Modern technology (without AI)	Modern technology (with AI)
Core goal	Diagnose/confirm disease (often after symptoms)	Measure disease-related retinal features quantitatively	Predict risk/prognosis early + automate stratification
Primary data	Vitals + labs + clinical history; specialist tests	Retinal imaging (fundus/OCT/OCTA) + predefined indices	Retinal imaging + learned representations + clinical metadata
Retinal role	Limited/optional; manual observation if used	Quantitative biomarkers (e.g., RNFL thickness, vessel caliber, capillary density)	End-to-end prediction (risk score, staging, progression, multi-disease inference)
Typical tools	BP cuff, lipid panel, ECG; MRI/PET for neuro	Fundus camera, OCT, OCTA, UWF imaging; software-based measurements	Deep learning (CNN/ViT), multimodal fusion, self-supervised/foundation features
Interpretation	Clinician-driven; subjective variability	Rule/threshold- and metric-driven; still interpretation-heavy	Automated decision support + explainability overlays for clinicians
Sensitivity to subtle patterns	Low–moderate (depends on clinician + tests ordered)	Moderate (limited to what metrics capture)	High (detects distributed, weak signals across pixels and modalities)
Screening scalability	Moderate; constrained by clinics & specialists	High for acquisition, moderate for interpretation	High: acquisition + automated triage supports population screening
Cost per screening cycle	Moderate–high (specialist + tests; imaging often expensive)	Moderate (retinal imaging scalable; analysis tools cost varies)	Moderate upfront (model/dev), low marginal cost per screen at scale
Invasiveness	Often moderate (blood draws, CSF in neuro cases)	Non-invasive imaging	Non-invasive imaging + automated analytics
Repeatability for monitoring	Limited by cost and access	High (serial imaging feasible)	Very high (continuous/longitudinal prediction and automated tracking)
Cross-disease integration	Siloed (cardio vs. neuro vs. metabolic)	Mostly modality- or disease-specific analysis	Multi-task models can jointly assess vascular + neurodegenerative risk
Multimodal fusion	Manual fusion of reports and tests	Limited: separate metrics per modality	Strong: fundus + OCT + OCTA + clinical data fusion (early/late/attention fusion)
Explainability	Clinical reasoning is explainable but subjective	Metrics are interpretable but incomplete	XAI tools (saliency/SHAP/attention) + uncertainty estimation
Generalization across devices/sites	Human variability; depends on local protocol	Device/operator variability affects metrics	Requires external validation + domain adaptation/robust training
Bias/fairness risks	Access bias + subjective assessment	Dataset/device bias persists	Demographic/device bias evaluation; fairness auditing possible
Output format	Diagnosis/referral notes; risk scores from calculators	Quantitative retinal metrics + descriptive reports	Risk/prognostic scores, triage category, progression likelihood, alerts
Deployment setting	Hospitals/clinics	Vision centers; tele-ophthalmology possible	Primary care + tele-screening + community programs (specialist-in-the-loop)
Key limitations	Late detection; expensive; fragmented workflows	Metrics may miss subtle signatures; standardization challenges	Data/validation/regulatory needs; trust/interpretability demands

**Fig. 1:**
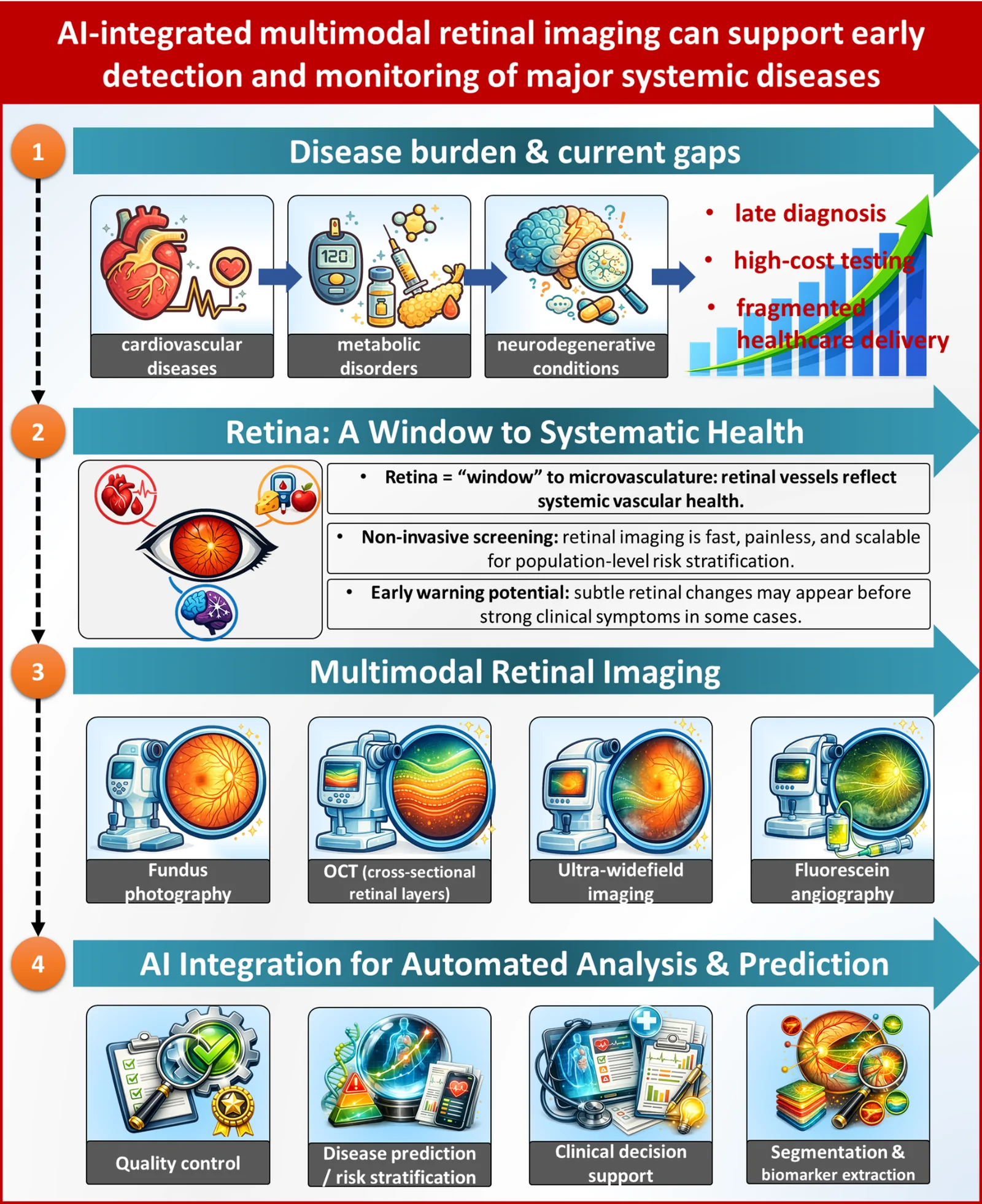
AI-enabled multimodal retinal imaging pipeline for systemic disease screening, risk stratification, and clinical decision support

In this regard, the retina provides a highly useful “window” into systemic health (see Fig. 1). The retinal microvasculature has similarities in structure and function to cerebral and systemic microcirculation, and neuroretinal tissues reflect processes of relevance to brain health. Notably, retinal imaging is non-invasive, relatively inexpensive, repeatable, and scalable to high-throughput analysis, making it highly appealing for population-level screening and longitudinal risk assessment [19–22]. In the past decade, advances in imaging have greatly expanded the scope of what can be assessed from the eye: color fundus photography can assess vascular morphology and visible lesions; OCT can quantify structural features such as RNFL and macular thickness; OCTA can assess capillary density and perfusion; and fluorescein/ultra-widefield angiography can map leakage and peripheral ischemia [23, 24]. Newer modalities such as hyperspectral imaging, adaptive optics, oximetry, and fluorescence lifetime imaging, provide metabolic and cellular-level information. Critically, these modalities are complementary: structural, vascular, and metabolic signatures provide different but synergistic views of early pathology, motivating multimodal fusion rather than reliance on any single modality [25, 26].

However, the task of teasing out meaningful systemic information from such a rich source of retinal data is not straightforward. However, human interpretation and single-feature analysis could potentially ignore subtle global patterns and still be difficult to normalize for different instruments, observers, and populations. It is in this domain that AI-assisted retinal analysis has been revolutionary: this domain is rapidly moving from retina-focused ophthalmology to the prediction of systemic diseases, using deep learning, multimodal fusion, and explainable AI to detect patterns that might be difficult to discern by human grading [27–29]. In terms of technical development, the journey has moved from manually engineered feature analysis to convolutional neural networks and vision transformers, increasingly enabled by self-supervised or foundation models pretraining that can be shared across diseases and imaging modalities. Multimodal learning architectures such as early fusion, late fusion, cross-modal attention, and hybrid models integrating clinical metadata enable joint inference from fundus + OCT + OCTA (and beyond), aligning with the biological reality that systemic disease signatures manifest across multiple retinal compartments. At the same time, clinical adoption depends on trust, fairness, and generalizability: explainability (e.g., saliency and attribution methods) and robustness evaluation (domain shift, dataset bias, external validation) are now essential considerations for safety-critical deployment [30–32].

### Scope and rationale of this review

This review focuses explicitly on AI-integrated multimodal retinal imaging for early detection, prognosis, and risk stratification of systemic vascular disorders (e.g., hypertension, stroke risk, coronary disease, systemic microvascular complications) and neurodegenerative disorders (e.g., Alzheimer’s, Parkinson’s, cognitive decline). Beyond cataloguing algorithms, we emphasize an end-to-end translational pathway: (i) biological and pathophysiological links between retinal changes and systemic disease; (ii) modality-specific and multimodal retinal imaging technologies; (iii) AI methodology (including multimodal fusion, explainability, and generalization); (iv) disease-focused retinal biomarkers; and (v) integration with broader precision-medicine data streams (neuroimaging, genomics, wearables, longitudinal prediction), aligned with real-world implementation requirements.

### How this review differs from prior reviews

However, the existing literature has made significant progress but tends to be siloed by disease type, modality, or AI vs. non-AI approaches. For the cardiovascular disease applications, there are reviews that specifically focus on deep learning with fundus images for cardiovascular risk prediction and scoping/systematic mappings of fundus + deep learning for cardiovascular markers/outcomes [33–35]. Other reviews synthesize AI approaches for CVD risk and fundus/OCTA-related signals but tend to be more cardiovascular disease-focused.

For neurodegenerative diseases, there are reviews that specifically focus on retinal imaging biomarkers for neurodegenerative diseases in general, often without a systematic AI systems perspective; others tend to focus on OCT/OCTA biomarkers for Alzheimer’s disease or quantify the AI-assisted retinal imaging diagnostic accuracy through systematic review/meta-analysis [36–38]. Non-AI modality-focused reviews tend to emphasize microvascular links and standardization issues but only gesture towards AI as a future direction. Lastly, more general “systemic diseases” AI-retina reviews tend to emphasize the opportunity landscape but may not necessarily integrate multimodal fusion architectures, robustness/fairness, and precision medicine into a unified translational framework [39–42]. In contrast, our review aims to weave together the pathways of vascular and neurodegenerative disease on a systems level perspective of multimodal + AI systems, making connections between biology → imaging physics → multimodal learning → risk stratification, with a special focus on generalization, interpretability, and implementation as design considerations.

## Literature search strategy and study selection

For enhancing the methodological transparency of the research process, a systematic literature search using a narrative approach was undertaken to find relevant literature on multimodal retinal imaging using AI technology for the assessment of systemic vascular diseases and neurodegenerative conditions. The literature search was done by using leading scientific databases such as PubMed/MEDLINE, Scopus, Web of Science, IEEE Xplore, and Google Scholar. Other literature was obtained by performing citation chaining from the relevant review papers, highly cited original papers, and recent foundation model/multimodal retinal imaging papers.

Keywords employed for the literature search included the following combinations of the below mentioned terms: “retinal imaging,” “fundus photography,” “optical coherence tomography,” “OCT,” “OCTA,” “retinal vascularization,” “oculomics,” “artificial intelligence,” “machine learning,” “deep learning,” “self-supervised learning,” “foundation model,” “multimodal learning,” “explainable AI,” “cardiovascular diseases,” “hypertension,” “high blood pressure,” “stroke,” “coronary heart disease,” “diabetes,”

### Study selection process

In all, a total number of 721 articles initially identified through database searches and citations. Following exclusion of 126 duplicate articles, 595 records underwent screening by titles and abstracts. At this stage, 288 records were found to be irrelevant as they did not fit with the themes of the paper which included retinal imaging, AI/ML, multimodal imaging, systemic vasculopathy, neurodegeneration, and translational retinal biomarkers. In the third step of inclusion and exclusion, full-text assessment was performed on 307 articles, out of which 110 were excluded. This left 197 articles to be reviewed.

### Inclusion criteria

Studies were included if they met one or more of the following criteria:

- Imaging techniques for retinal imaging, such as fundus photography, OCT, OCTA, fluorescein angiography, ultra-widefield imaging, hyperspectral imaging, retinal oximetry, adaptive optics, or fluorescence lifetime imaging.
- AIs and/or ML techniques (e.g., deep learning, self-supervised learning, foundation models), and/or multimodal approaches, or explainable AI applied to retinal images.
- Systemic vascular/microvascular or metabolic conditions, such as hypertension, stroke, coronary artery disease, diabetes-associated pathology, chronic kidney disease, or systemic microvascular impairment.
- Neurodegenerative/neuroinflammatory conditions, such as Alzheimer’s disease, Parkinson’s disease, multiple sclerosis, cognitive impairment, brain aging, or associated neurological consequences.
- Relevant clinical information about model development, validation, performance, multimodal combination, biology, translational aspects, implementation obstacles, health economic implications, etc.
- Peer-reviewed original research papers, systematic/review papers, meta-analyses, or technical/consensus papers relevant to this review.

### Exclusion criteria

Articles were excluded if they met any of the following criteria:

- They were not related to retinal imaging or ocular biomarkers.
- They dealt solely with conventional ophthalmology disease diagnosis without any vascular or neurodegenerative relevance.
- They were devoid of AI/ML, automated image processing, quantitative retinal biomarkers, or multimodal imaging relevance.
- They were strictly scientific imaging research without any clinical or translational value.
- They were lacking adequate methodological detail, validation data, or interpretability for meaningful integration.
- They were duplicates, overlap with existing datasets without adding new insights, editorial letters without technical or clinical content, or conference proceedings without any full-text information.
- They were not in the English language or insufficiently informative for review.

### Bias and evidence quality considerations

Since this paper is a narrative review rather than a formal systematic review or meta-analysis, a risk of bias assessment was not numerically performed on the included studies. The included literature has been reviewed in terms of common limitations that may exist in the use of AI in medical imaging research. In particular, factors such as the inclusion of independent external validation; multi-center data sets; prospective versus retrospective study designs; adequate patient samples and diversity; clear clinical labels; model performance metrics; model calibration; model explainability; and the presence of confounding factors (e.g., age, diabetes, hypertension, eye conditions, image quality, type of imaging machine, etc.) were considered. Extra caution was taken with those papers that had smaller sample size; single-center patient populations; enriching the case-control group; inadequate diversity among participants; lack of independent external validation; shared data sets; and no prospective clinical trials. Since artificial intelligence in retinal imaging is at high risk for over-fitting, publication bias, and domain shifts, extra emphasis was placed on translatability, performance, validity, generalizability, interpretability, and usability in clinical practice.

## Biological and pathophysiological basis linking retina to systemic disease

### Retinal microvascular architecture and systemic circulation

**Fig. 2:**
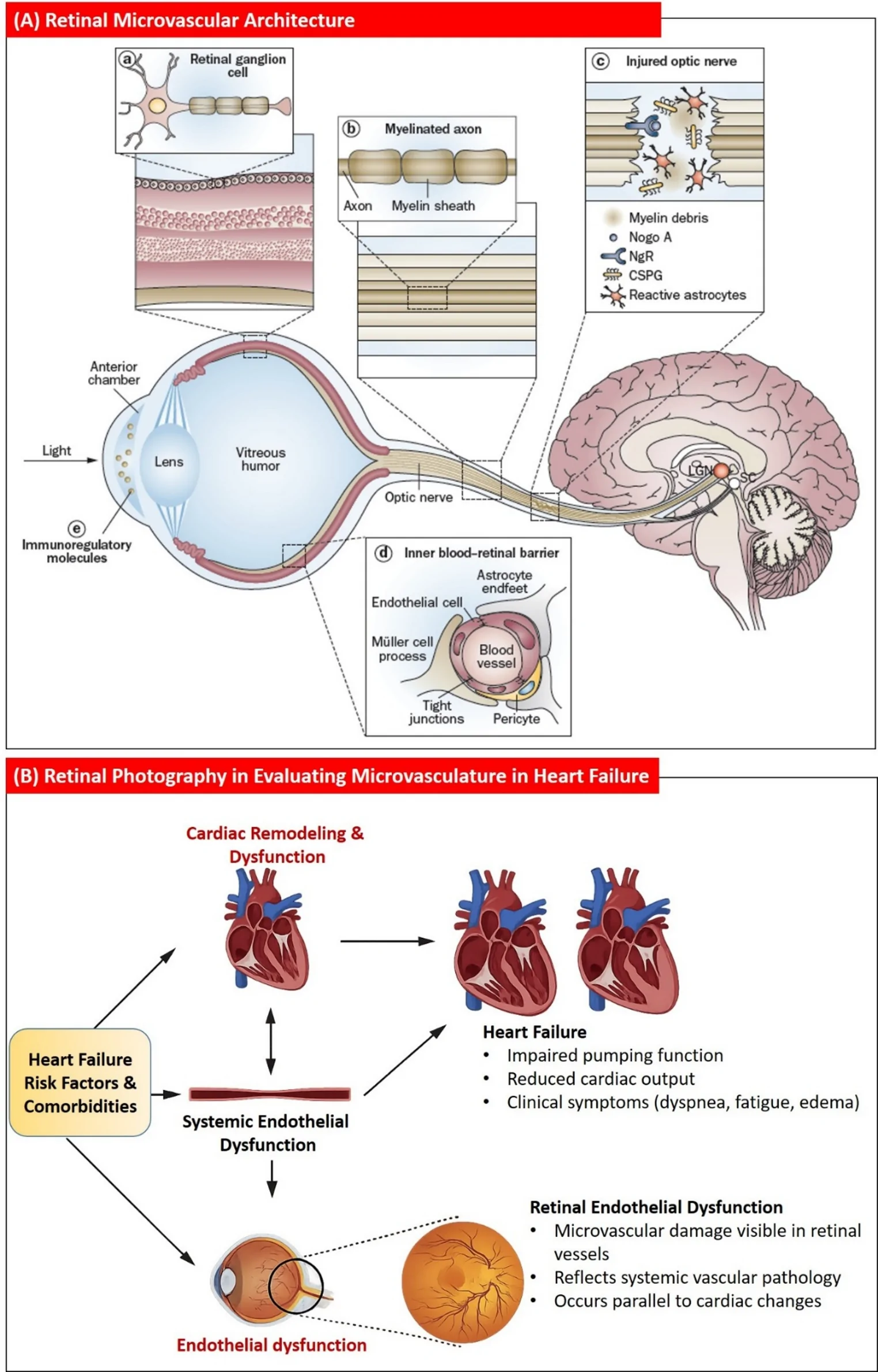
**(A)** Retinal microvascular architecture and inner blood–retinal barrier structure taken from [47] with the permission of Springer. **(B)** Retinal photography as a non-invasive indicator of systemic endothelial dysfunction and heart failure progression

The retina (the light-sensitive tissue at the back of the eye) is full of small blood vessels, small arteries, very small capillaries, and small veins, which can be easily visualized by eye imaging. This is important because these small vessels are similar to the microcirculation in organs like the brain, heart, and kidneys, but these organs are much more difficult to image directly (see Fig. 2A and B). If a person has ongoing conditions such as high blood pressure, diabetes, high cholesterol, or inflammation, the inner lining of blood vessels (endothelium) can become sick and less functional in regulating the widening and narrowing of vessels [43–45]. Over time, vessels may become stiffer, some vessels may become narrower, and the smallest capillaries may decrease in number or become damaged. These changes can be visualized in the retina as narrower small arteries, wider small veins, more tortuous vessels, and changes in vessel branching. Because these changes are indicative of underlying stress on the body’s circulation, the retinal microvessels offer a direct and non-invasive window into the body’s overall vascular health and can often predict risk earlier, often before serious symptoms or organ damage occur [46].

### Neuroretinal structure as a proxy for brain health

The retina is more than an “eye tissue,” it is a part of the central nervous system, so alterations in the retina’s neurons can indicate what is happening in the brain. The most relevant layers of the retina for this are the Retinal Nerve Fiber Layer (RNFL) and the ganglion cell complex (commonly assessed around the macula as the ganglion cell-related thickness). These layers contain the axons and cell bodies of retinal ganglion cells, which directly form the optic nerve and carry visual information to the brain [48–50]. When neurodegenerative processes begin, neurons and their connections (synapses) slowly weaken and are lost; similar “wear and tear” can be detected as thinning or structural change in these retinal layers. Because of this, OCT (Optical Coherence Tomography) measurements of RNFL and ganglion cell layers are increasingly used as a practical, non-invasive way to track neurodegeneration over time.

Importantly, several brain disorders show parallel patterns of retinal neuronal change, for example, synaptic degeneration parallels have been reported in conditions such as Alzheimer’s and Parkinson’s disease, making the neuroretina a useful proxy for brain health. Overall, neuroretinal structure provides a repeatable and scalable window into neuronal integrity, supporting early risk signals and longitudinal monitoring, especially when combined with other retinal vascular and imaging biomarkers [51, 52].

### Metabolic, inflammatory, and hypoxic signatures

Metabolic stress, inflammation, and hypoxia impart “footprints” to the retina due to the high energy requirements and delicate microcirculatory regulation of retinal cells. In metabolic disease (particularly chronic hyperglycemia), hyperglycemia-induced glycemic damage occurs through protein/lipid modification (AGEs), osmotic damage, and microvascular damage, which compromises the support cells of capillaries and the blood-retina barrier. Simultaneously, oxidative stress (elevated levels of reactive oxygen species) causes mitochondrial, lipid, and DNA damage to both vascular and neuronal retinal cells, accelerating endothelial dysfunction and capillary loss [53–55]. These metabolic damages frequently overlap with neuroinflammation, in which activated glial cells and pro-inflammatory cytokines synergistically enhance neuronal stress, vascular dysfunction, and subtle neuroretinal thinning. As a consequence of inefficient perfusion, local hypoxia may also emerge; hypoxia-inducible pathways (such as HIF-1) induce the expression of pro-angiogenic factors, including VEGF, which increases vascular permeability and, in later stages, drives ischemia-mediated remodeling. These pathologies may manifest clinically as early microvascular and tissue alterations visible on retinal imaging, capillary density/perfusion deficits on OCTA, retinal edema or layer abnormalities on OCT, and microlesions (microaneurysms, hemorrhages, exudates) on fundus photography, offering a useful, non-invasive surrogate for the systemic metabolic and inflammatory burden [56–58].

### Temporal progression: retinal changes before clinical disease

Many systemic vascular and neurodegenerative disorders have a slow course, so that the retina may begin to demonstrate measurable changes before a patient reaches a definite “clinical diagnosis.” In this preclinical phase, the first signs are often subtle and multifocal, small changes in vessel morphology (caliber, tortuosity, branching), early decreases in capillary flow on OCTA, or mild thinning of neuroretinal layers (e.g., RNFL/ganglion cell layers) on OCT. Such changes may not be symptomatic, but could represent early endothelial dysfunction, low-grade inflammation, metabolic insult, or incipient neurodegeneration [59]. The power lies in longitudinal observation: if retinal images are obtained serially over months to years, the course (rate and direction of feature change) may be more revealing than a static image. This is where longitudinal cohort data becomes valuable – large cohorts prospectively followed over time can help confirm which retinal patterns emerge earliest, which patterns predict future disease, and to what degree they correlate with later outcomes. In practice, this underlies a transition from “disease detection” to “risk and progression detection early,” allowing for earlier prevention, closer surveillance of high-risk patients, and more tailored intervention timing [60, 61].

## Multimodal retinal imaging technologies

### Color fundus photography

Color fundus photography is a very efficient method of obtaining a 2D color image of the retina, including the optic disc, macula, and retinal vessels. In terms of clinical utility, it is a very valuable tool because it allows for the direct visualization of vessel morphology (e.g., changes in vessel appearance) and typical lesions of the retina, such as hemorrhages and hard exudates, which are indicative of microvascular damage due to systemic diseases such as diabetes and hypertension. Because this test is relatively inexpensive and can be easily repeated, it is a natural fit for tele-ophthalmology applications of screening large numbers of people with minimal input from specialists [62].

AI improves fundus photography by transforming these images from “visual inspection” to standardized, high-throughput analyses. AI, in the form of deep learning algorithms, can automatically (i) assess image quality (to prevent unreadable images), (ii) assess and grade lesions (such as hemorrhages/exudates), and (iii) assess vessel-related features at scale. Crucially, there are large datasets in fundus photography that are amenable to AI, making it feasible to deploy AI models across screening programs. In practical screening programs, this would translate to faster processing, fewer subtle abnormalities missed, and a specialist-in-the-loop system where AI-pre prioritized high-risk patients are confirmed and treated by specialists [63, 64].

### Optical Coherence Tomography (OCT)

Optical Coherence Tomography (OCT) is a non-invasive retinal imaging modality that offers high-resolution, cross-sectional “slice” views of the retina, enabling measurements of tissue structure rather than simple visual assessment. In systemic and neurodegenerative risk settings, OCT is particularly useful as it allows for the structural quantification of important neuroretinal layers, particularly RNFL and macular thickness, which are extensively used as neurodegenerative biomarkers and can be monitored longitudinally for subtle changes. AI significantly enhances the scalability and utility of OCT by automating the most time-consuming and challenging parts of the process [65, 66]. Deep learning algorithms can automatically handle quality control, layer segmentation, and thickness mapping on a large scale of scans, which are prone to human variability. While going beyond simple measurements, AI can identify weak and distributed patterns in OCT reflectivity and layer maps that are difficult to detect using rule-based systems, providing decision-support outputs and, if required, explainability overlays for clinician acceptance. Most importantly, OCT is even more effective when combined with other retinal imaging modalities by AI: multimodal fusion (fundus + OCT + OCTA, with clinical metadata) is consistent with the observation that systemic disease patterns are present in all retinal compartments, which improves early risk assessment and monitoring [67, 68].

### Fluorescein and wide-field angiography

Fluorescein angiography (FA) is a dye-based retinal imaging technique where fluorescein dye is injected (usually intravenously) and rapid serial images are taken as the dye passes through the retinal vasculature. This “movie” type of imaging is particularly useful for demonstrating vascular leakage (due to damaged or abnormal vessels) and areas of poor perfusion/non-perfusion, which are important markers of active or advanced microvascular disease [69–71]. When done with wide-field/ultra-widefield imaging systems, the imaging fields extend beyond the central retina to the peripheral retina, allowing the clinician to detect peripheral ischemia that may not be visible within standard fields of view, which is important for risk assessment and treatment planning in severe retinal vascular disease (e.g., proliferative diabetic retinopathy, retinal vein occlusions, vasculitis). In practice, FA and wide-field angiography are often used to map severe vascular disease and guide interventions (e.g., laser therapy) because they provide information on where the leakage and ischemia are most active. Value addition comes from the ability of AI to transform angiography from a highly specialized interpretive procedure into quantified and reproducible results. The AI system has the capability to automatically segment and quantify non-perfusion regions, identify and grade the intensity of leakage, and identify high-risk regions for early prioritization. Additionally, in a multimodal processing environment, AI has the capability to integrate angiography signals with OCT/OCTA and fundus findings to provide a comprehensive system-level vascular severity profile [72, 73].

### Emerging and experimental modalities

Emerging and experimental retinal imaging modalities seek to go beyond “structure and vessels” and provide metabolic, oxygenation, and cellular-level information that may become altered very early in systemic vascular and neurodegenerative disease. Hyperspectral retinal imaging captures the retina over a broad range of very narrow wavelengths, allowing for more detailed tissue “spectral fingerprints” that can be quantitatively related to blood/chromophore concentration and metabolic-related optical effects. Adaptive optics cellular imaging corrects for ocular aberrations to image retinal microstructure at near-cellular resolution, facilitating the detection of subtle neuronal or microvascular changes that may be obscured by conventional imaging [74, 75]. Retinal oximetry measures oxygen saturation in retinal vessels, providing a functional measure of oxygen delivery/consumption that is directly relevant to hypoxia-mediated pathology. Fluorescence lifetime imaging measures the fluorescence decay properties of endogenous fluorophores, offering a highly sensitive window into cellular metabolism and stress responses. Taken together, these technologies introduce metabolic and cellular axes to the multimodal retinal imaging toolkit and can be used in conjunction with OCT/OCTA and fundus imaging, rather than instead of them [76, 77]. AI is especially helpful in this respect because these technologies produce complex high-dimensional data. AI models can improve denoising and artifact correction, automate segmentation/quantification, offer spectral unmixing (hyperspectral imaging), and identify subtle patterns of disease even when these patterns are small and diffuse. Finally, AI allows the combination of these experimental signals with OCT/OCTA/fundus data to provide a system-level risk profile. A concise comparison of multimodal retinal imaging technologies and the ways AI strengthens each modality is summarized in Table 2.

**Table 2:** Multimodal retinal imaging technologies: signals, readouts, use-cases, and AI value-add

Modality	What it captures	Typical readouts (examples)	Best use-case	How AI helps (practical value)
Color Fundus Photography	2D color view of retina, optic disc, macula, and visible vessels/lesions	Vessel morphology; hemorrhages; hard exudates	Population screening and first-line triage (including tele-screening)	Automated image quality checks; lesion detection/grading; vessel feature extraction; scalable triage workflows
OCT (Optical Coherence Tomography)	High-resolution cross-sectional retinal structure	RNFL and macular thickness; layer-level structural quantification	Neuroretinal integrity monitoring; early structural biomarkers; longitudinal tracking	Automated layer segmentation and thickness maps; standardized reporting; subtle pattern detection beyond simple rules
OCTA (OCT Angiography)	Dye-free microvascular flow/perfusion maps across retinal plexuses	Capillary density and perfusion indices; microvascular dropout/non-perfusion surrogates	Early microcirculatory impairment and ischemia-related risk signals; progression monitoring	Artifact reduction; robust segmentation/quantification; detection of subtle perfusion loss; fusion with OCT/fundus features
Fluorescein and Wide-Field Angiography	Dye-based dynamic vascular imaging; wide-field extends to peripheral retina	Leakage; peripheral ischemia; mapped non-perfusion regions	Deep vascular phenotyping and treatment planning when leakage/ischemia mapping is needed	Automated leakage/non-perfusion quantification; severity scoring; faster triage; consistent longitudinal comparison
Emerging and Experimental Modalities	Advanced functional/cellular/metabolic signals (e.g., hyperspectral, adaptive optics, oximetry, fluorescence lifetime)	Spectral signatures; near-cellular microstructure; oxygenation estimates; metabolic stress proxies	Research and early-signal discovery; complements OCT/OCTA/fundus in multimodal studies	Denoising and artifact correction; high-dimensional feature learning (e.g., spectral unmixing); automated quantification; multimodal fusion

## AI methodologies in retinal image analysis

### Evolution from classical machine learning to deep learning

The classical approach to retinal image analysis involved feature engineering, where the first step involved designing features that could be measured (e.g., vessel size/tortuosity, texture features, number of lesions, optic disc/cup shape, OCT layer thickness), and then these hand-designed features were used as input to “classical” ML algorithms like logistic regression, support vector machines (SVM), k-nearest neighbors (kNN), random forests (RF), and gradient boosting. This is a good approach when the amount of data is small and when the ML model needs to focus on well-defined biomarkers; but in many cases, it is limited by the quality of segmentation and the capabilities of the engineered features [78–81].

Deep learning introduced the paradigm of end-to-end learning, where models directly learned the most predictive features from pixels/voxels. Convolutional neural networks (CNNs) (such as VGG, ResNet, Inception, and EfficientNet series) have become the engine for fundus/OCT image classification and risk assessment due to their ability to identify subtle, distributed patterns that are difficult to manually encode. For pixel-level tasks, U-Net-like architectures (and their variants, Attention U-Net/DeepLab decoder networks) facilitate vessel/lesion/layer segmentation and quantification. More recently, Vision Transformers (ViT/Swin) have emerged as popular alternatives because attention networks are better at capturing global context and scale much better, particularly with the advent of multimodal learning and large-scale pretraining. The progression can thus be succinctly described as feature engineering → CNNs → Vision Transformers, allowing for greater sensitivity, scalability, and generalization performance when properly trained and validated [82–84].

### Self-supervised and foundation models

**Fig. 3:**
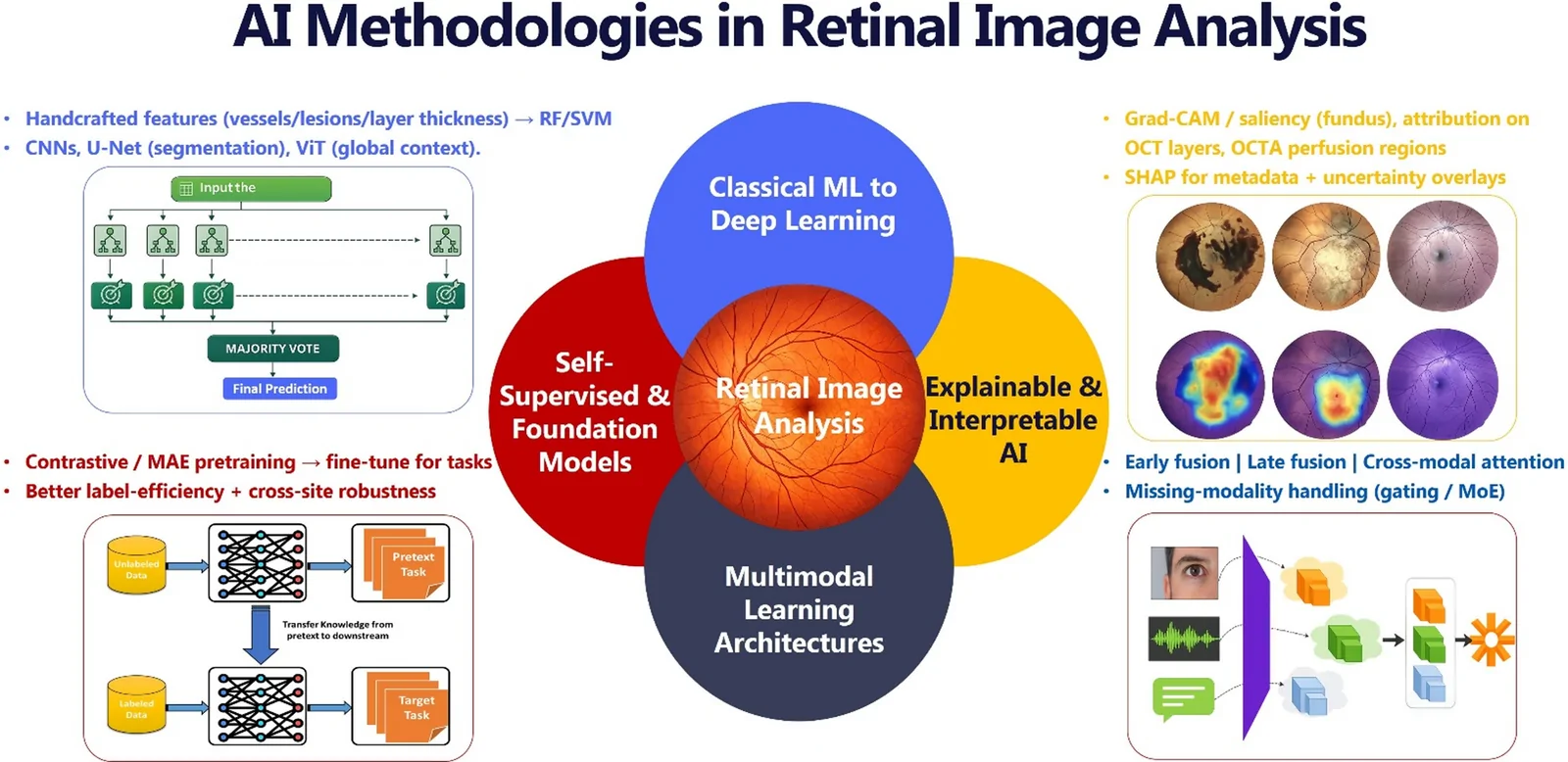
AI approaches in retinal image analysis: from classical ML to multimodal and explainable deep learning

Self-supervised learning (SSL) and foundation models address a key bottleneck in retinal AI: high-quality labels are expensive, but retinal imaging produces huge volumes of unlabeled data. In SSL, the model learns useful representations without manual labels by solving “pretext” tasks, then transfers that knowledge to downstream clinical tasks with far fewer annotated examples (see Fig. 3). Common SSL strategies include contrastive learning (e.g., learning to pull together different augmentations of the same image and push apart different images), and masked-image modeling (e.g., masked autoencoders, MAE) where the network reconstructs missing patches, both are highly effective for fundus photos and OCT/OCTA volumes [85–87].

Foundation models extend this idea: large CNN/Transformer backbones are pretrained on very large, diverse retinal datasets (often multi-center, multi-device), then adapted for specific tasks such as diabetic retinopathy grading, glaucoma risk, AMD detection, OCT layer segmentation, or OCTA perfusion quantification. Practically, they improve label efficiency (good performance with small labeled sets), robustness across devices and populations, and generalization to new sites (reduced domain-shift sensitivity). They also enable few-shot fine-tuning and lightweight adaptation using methods like adapters/LoRA, which is useful when data-sharing is constrained. Finally, multimodal foundation models can learn shared representations across fundus + OCT + OCTA (and clinical metadata), making them ideal for unified risk prediction rather than single-disease classification [29, 88–90].

### Multimodal learning architectures

Multimodal learning architectures are designed to jointly interpret fundus + OCT + OCTA (and sometimes clinical metadata) because systemic disease signatures do not appear in only one retinal compartment; structural, vascular, and perfusion cues are complementary. A common starting point is early fusion, where features (or embeddings) from different modalities are combined early e.g., concatenation of modality encoders followed by a shared predictor, so the model can learn cross-modality interactions from the beginning [91, 92]. Late fusion takes an ensemble-style approach: each modality has its own specialized network, and their outputs are combined at the decision level (weighted averaging, stacking, or learned gating), which is often more robust when modalities have different noise profiles or availability. More powerful designs use cross-modal attention transformers, where one modality can “attend” to informative regions/features in another, enabling richer alignment between vascular patterns (OCTA/fundus) and neuroretinal structure (OCT), and helping when one modality is partially missing or lower quality. Finally, graph neural networks can integrate clinical metadata with imaging embeddings (and even relational structure), supporting patient-level risk stratification where demographic, lab, or comorbidity context meaningfully modulates imaging signals [93–95].

### Explainable and interpretable AI

Explainable and interpretable AI (XAI) is a key area in retinal risk prediction because AI models could potentially impact decisions in retinal screening, referral, and long-term care, and therefore, there is a need for clinicians to interpret the reasoning behind a risk prediction. Practically, XAI techniques can be broadly categorized into three complementary categories. First, there are post-hoc visual techniques that provide image region explanations for the decision-making process, which include saliency/heatmaps like Grad-CAM and other attribution techniques. These are helpful in ensuring that the model is paying attention to biologically plausible structures (such as vessels, hemorrhages/exudates in fundus, RNFL/macular layers in OCT, and non-perfusion areas in OCTA) and not artifacts. Second, there are feature-level explanations for models that predict quantitative biomarkers or use engineered features; these include SHAP or permutation importance explanations that can help identify which inputs (such as vessel density, RNFL thickness, tortuosity, age, BP, and diabetes status) contributed most to a risk prediction [96]. Third, there are intrinsically interpretable models, where interpretability is designed into the model; these include attention-based multimodal fusion models where attention weights can provide information on modality/region contributions, or concept-based models that predict intermediate clinical concepts (such as “capillary dropout present”) before the final disease/risk prediction. Crucially, XAI needs to be tested and not assumed; explanations should be robust to small perturbations, consistent with expert intuition, and examined for spurious correlations (device marks, illumination, compression artifacts). When properly implemented, interpretability can enhance trust, facilitate error analysis, facilitate safer deployment across multiple sites, and help to provide a basis for translating retinal AI predictions into actionable clinical reasoning [97, 98].

## AI-derived retinal biomarkers for systemic vascular diseases

### Hypertension and blood pressure prediction

Hypertension is a condition that is “clinically quiet” yet, over time, it remodels the small vessels of the body. The field of retinal imaging is poised to show the impact of this remodeling because arterioles, venules, and capillaries can be photographed noninvasively and serially, providing insight into chronic vascular stress rather than a point measurement of blood pressure (BP). Retinal-AI work therefore follows two complementary goals: BP prediction (estimating systolic/diastolic values from images) and hypertension-risk screening (classifying likely hypertension or hypertensive end-organ impact). A key concept is that image-inferred BP may behave like a “vascular exposure” biomarker less sensitive to momentary measurement noise and potentially more indicative of long-term hypertension so it can complement cuff-based care rather than compete with it [99].

The contemporary trajectory of the area begins with large-scale oculomics. Poplin et al. trained deep learning models on UK Biobank/EyePACS fundus images and showed that systolic BP can be inferred from images, proving the existence of significant BP signal independent of gross lesions [100]. Rim et al. generalized this concept to the use of multiple systemic biomarkers (BP included), a paradigm shift from disease-specific diagnosis to the retina as a systemic sensor [101]. This led to the inclusion of structure and context by various groups. Arnould et al. combined quantitative retinal vascular analysis with OCTA analysis and conventional machine learning approaches to predict cardiovascular variables, showing the potential of engineered vessel description to improve machine learning models when applied to smaller datasets [102]. Continuing this line of work, White et al. used a model trained on large data sets in a real-world setting in Kenya, where blood pressure was simultaneously measured with fundus photographs, thus moving the research from proof of concept to transportability [103]. Squirrell et al. furthered the narrative toward clinical relevance by demonstrating that AI-predicted systolic BP from UK Biobank retinal images predicts future atherosclerotic cardiovascular disease events, indicating that the model reflects a risk-relevant vascular phenotype rather than a noisy point measurement [104].

**Fig. 4:**
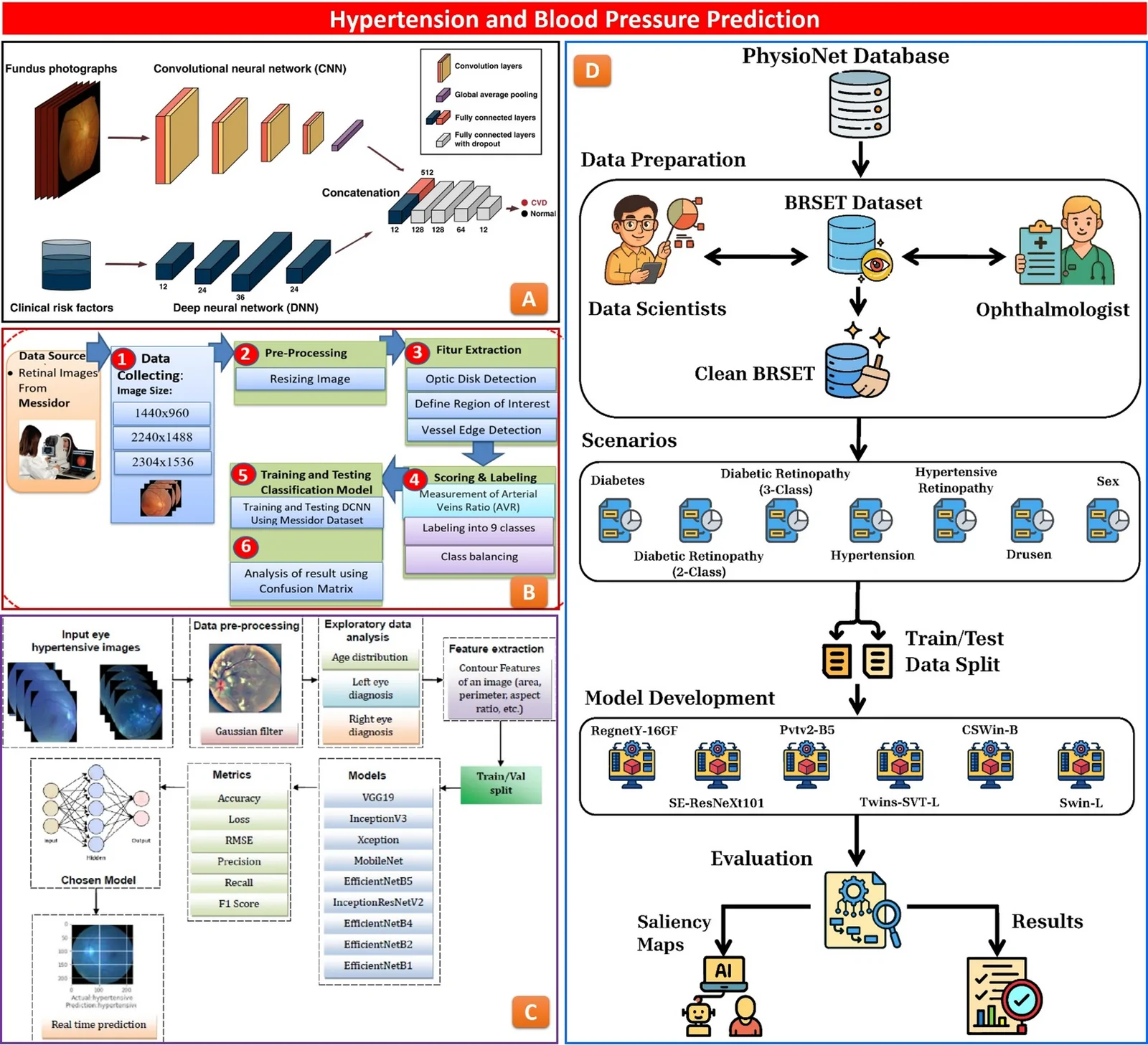
(**A**) CNN–DNN fusion model predicting CVD risk from fundus images + clinical risk factors, taken from [105] with the permission of Springer. (**B**) Data pipeline for extracting retinal biomarkers (optic disc, vessels, AVR) to support BP classification and CVD risk prediction, taken from [107] with the permission of Elsevier. (**C**) Model comparison framework evaluating accuracy, RMSE, precision, recall for hypertension detection and BP estimation, taken from [108] with the permission of Springer. (**D**) End-to-end BRSET/PhysioNet workflow for training and validating deep models to predict hypertensive retinopathy, hypertension presence, and BP levels, with saliency-based interpretation, replicated from [109] with the permission of MDPI

Concurrently, multimodal designs arose, Lee et al. integrated fundus images with clinical risk factors for CVD diagnosis (see Fig. 4A), and Baharoon et al. (HyMNet) demonstrated that integrating fundus images with basic demographics could enhance hypertension prediction, an essential step from “BP as a number” to “BP as risk” [105]. Simultaneously, disease-specific pipelines focused on hypertensive eye manifestations. Abbas et al. developed EfficientNet-based classification for hypertensive retinopathy [106], Triwijoyo et al. extended grading to multiple severity levels (see Fig. 4B) [107], and Kumar et al. investigated hybrid transfer-learning and morphological features for hypertensive vs. non-hypertensive fundus images (see Fig. 4C) [108]. More recent studies are also extending modality frontiers. Alooghareh et al. evaluated CNN/transformer backbones on a multi-label fundus image dataset that included hypertension (see Fig. 4D) [109], Ding et al. applied deep learning to OCTA images to detect hypertension based on capillary-level flow dynamics [110], and Bressler et al. described fundus-image-based BP biometry that predicts both systolic and diastolic BP and “clearly posits that it may indicate exposure to chronic hypertension” [99].

### Stroke risk and cerebrovascular disease prediction

Stroke is an acute clinical syndrome, but the underlying biology is typically slow: years of hypertension, diabetes, inflammation, and endothelial dysfunction alter small vessels, autoregulation, and “silent” brain injury. The retina is particularly valuable in this regard because it is the only location where the human microvasculature can be visualized noninvasively in detail, and the retinal vasculature has strong developmental and physiological analogies to cerebral small vessels. This makes for an attractive prevention strategy: use retinal images as a low-cost, repeatable “vascular audit,” and use AI to quantify patterns that are too subtle for visual grading either to (i) predict future stroke risk, or (ii) identify subclinical cerebrovascular phenotypes that would otherwise require MRI. Generally, the community is shifting from “retinal traits predict stroke” to “retinal-AI models that are useful in real-world practice.”

This evolution can be traced through interrelated case studies. Early studies formulated the retina as a stroke biomarker discovery domain, comparing approaches to extract stroke-relevant retinal information from fundus imaging [111]. A major conceptual link then developed with the notion of “retinal biological age”. Zhu et al. trained a deep network to predict age from fundus images and found that the retinal age difference (retina-predicted age minus chronological age) predicted incident stroke, implying that retinal AI can encode the cumulative vascular effects of ageing over and above snapshot vital signs [112].

**Fig. 5:**
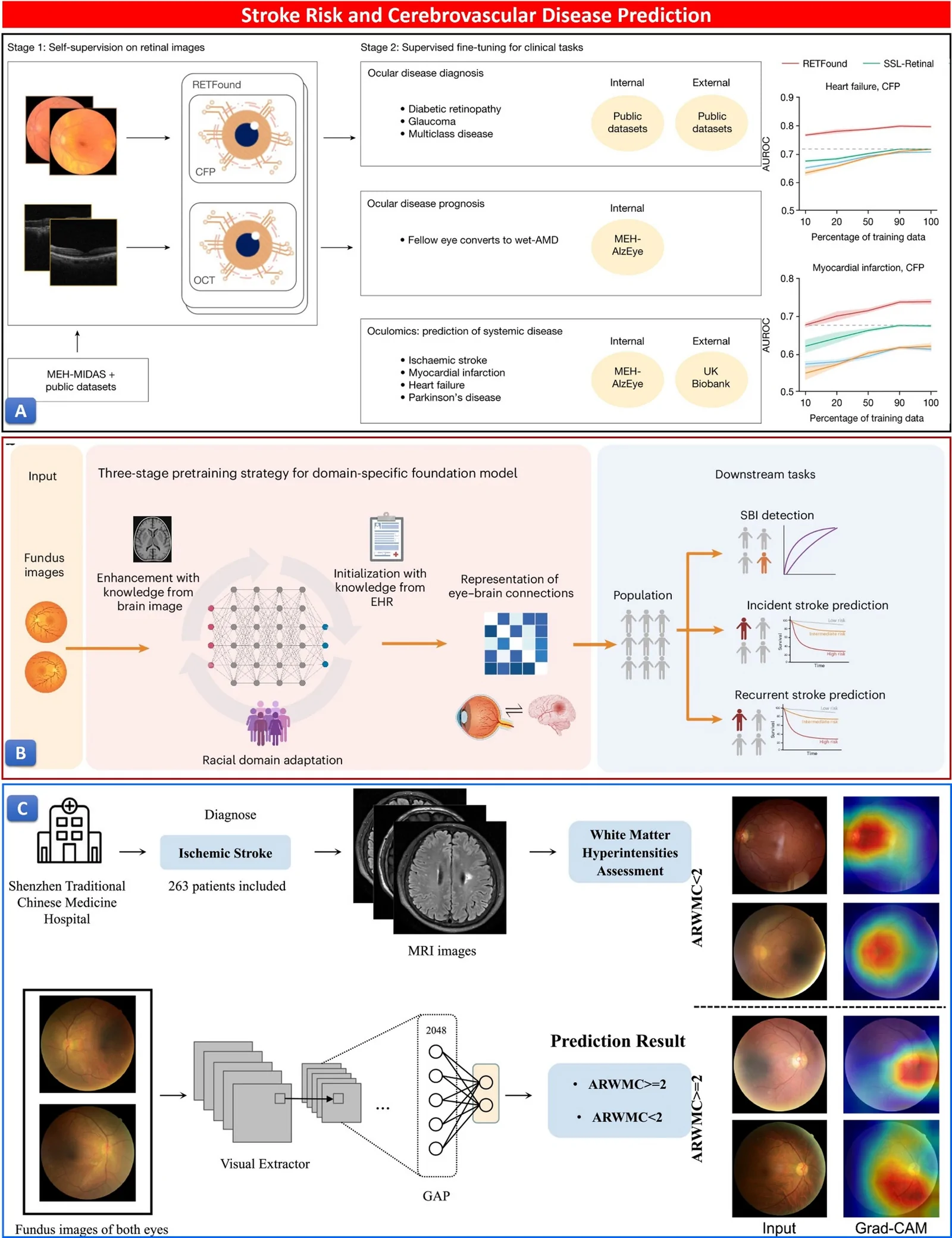
(**A**) Self-supervised retinal foundation model (RETFound) predicting stroke, myocardial infarction, heart failure, and other systemic diseases from fundus/OCT images, replicated from [29] with the permission of Springer. (**B**) Domain-adapted pretraining framework integrating brain imaging and EHR knowledge to predict incident and recurrent stroke risk at population level, taken from [114] with the permission of Springer. (**C**) Clinical pipeline using fundus images to predict ischemic stroke severity and white matter hyperintensities (ARWMC score ≥ 2 vs. < 2) with Grad-CAM interpretation, taken from [117] with the permission of Elsevier

This, of course, implies more nuanced phenotyping. Yusufu et al. extracted a full retinal vascular profile from fundus photos in the UK Biobank, discovering new vascular biomarkers predicting incident stroke and demonstrating predictive accuracy from retinal vasculature features [113]. Contemporaneously, model architecture evolved from hand-engineered vasculometry to deep learning and foundation models. RETFound (self-supervised pretraining on large-scale unlabeled retinal data) demonstrated generalizable representations that can be transferred to systemic outcomes such as ischaemic stroke prediction, illustrating the utility of label-efficient pretraining, as illustrated in Fig. 5A [29]. The most explicit “eye–brain” foundation step is DeepRETStroke, pretrained on 895,640 retinal photographs, validated across multi-country datasets, and designed to detect silent brain infarction and refine both incident and recurrent stroke risk directly connecting retinal signals to a subclinical cerebrovascular phenotype that usually needs brain imaging, as depicted in Fig. 5B [114]. Meanwhile, OCTA-based studies strengthen the mechanistic link to cerebral small vessel disease (CSVD). OCTA metrics correlate with MRI markers such as deep white matter lesions, perivascular spaces, and lacunes (Fu et al.) [115], while swept-source OCTA measures of deep capillary density relate to lesion burden (Geerling et al.) [116].

This retina-to-MRI bridge is extended by Zhuo et al., who used a deep learning approach leveraging retinal vessels to identify ischemic stroke patients with high white-matter hyperintensity burden using MRI as the reference, as shown in Fig. 5C [117], and by Pachade et al., who demonstrated that OCTA-derived microvascular density features (with self-supervised learning) can support stroke-related discrimination [118], collectively building continuity from risk prediction → subclinical brain injury → clinical stroke phenotypes.

### Coronary artery disease and atherosclerosis prediction

Coronary artery disease and atherosclerosis represent systemic, chronic pathologies in which lipid accumulation, inflammation, and endothelial dysfunction cumulatively stiffen and narrow arteries, while microvascular beds compensate via remodeling and rarefaction. The retina provides a highly informative model of these diseases, providing a noninvasive, reproducible window into microvascular health that is mechanistically and causally connected to the systemic circulation. Artificial intelligence adds value through improved scalability and sensitivity. Rather than focusing primarily on lesions, deep learning algorithms are able to discern subtle geometric, textural, and neurovascular patterns of vessel morphology that encode accumulated vascular “wear and tear,” thus facilitating an oculomics approach to atherosclerotic risk that might complement traditional lipid analysis and risk factor evaluation. A recent scoping review article summarizes the current state of deep learning on fundus images for inferring cardiovascular risk factors and disease risk, while also pointing out common pitfalls such as domain shift and the lack of prospective validation.

This path begins with the assessment of feasibility. Poplin et al. showed that deep learning can predict a number of cardiovascular risk factors, including systolic blood pressure and smoking status, as well as indicators of significant adverse cardiac events from fundus photographs, thus making a claim that systemic vascular risk is encoded in the retinal images [100]. Next, groups shifted from “risk factors” to subclinical atherosclerosis by predicting coronary artery calcium, a strong imaging proxy of plaque burden. Rim et al. introduced a retinal-predicted CAC score (RetiCAC) for cardiovascular risk stratification [119], and subsequent work (e.g., PRECISED cohort analyses) validated fundus-based prediction of high coronary artery calcium thresholds [120].

**Fig. 6:**
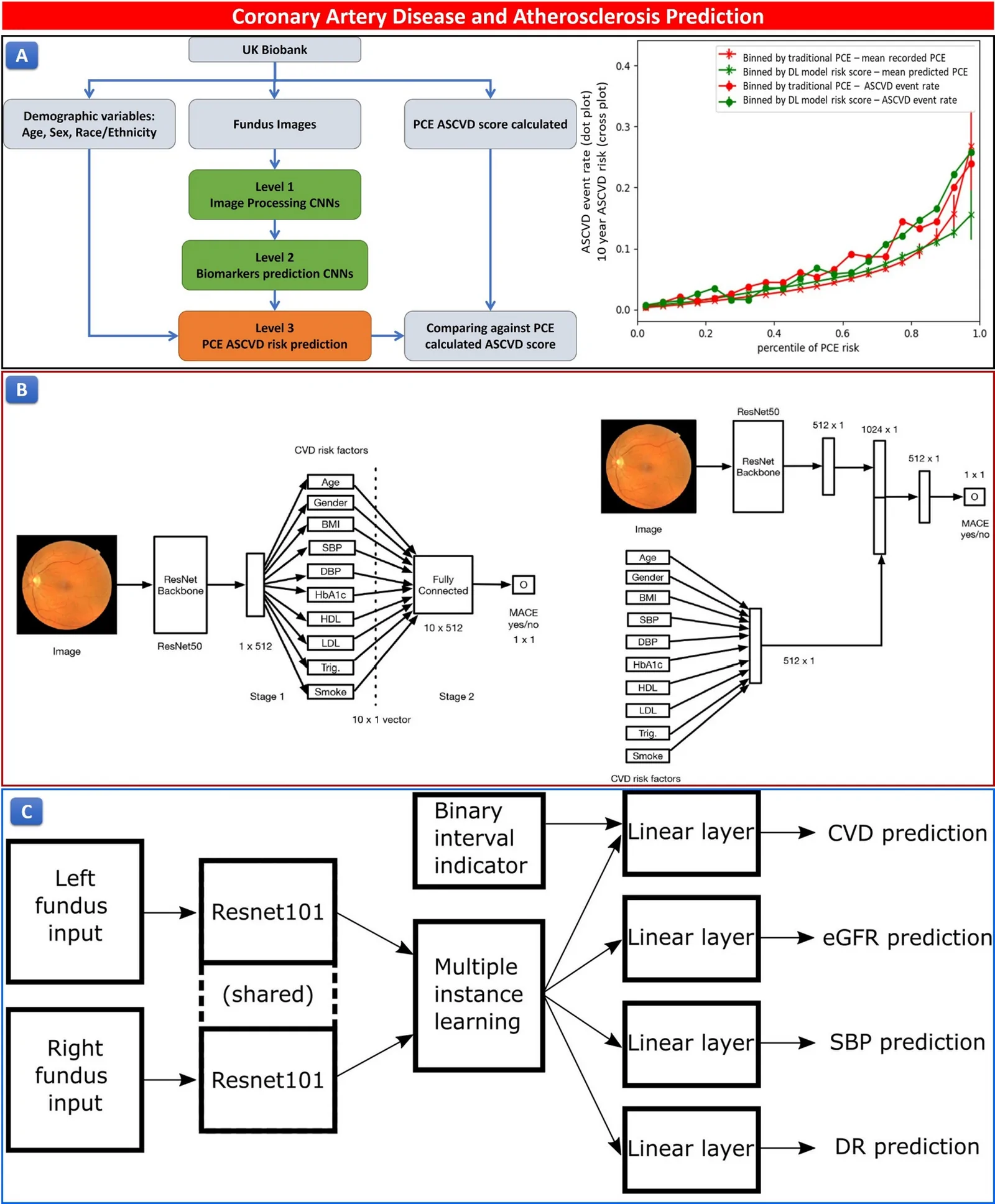
(**A**) Deep learning framework using UK Biobank fundus images to predict 10-year ASCVD (atherosclerotic cardiovascular disease) risk, compared against traditional PCE scores, replicated from [121] with the permission of Elsevier. (**B**) Multimodal CNN model combining retinal images and clinical risk factors to predict major adverse cardiovascular events (MACE) and coronary artery disease risk, taken from [122] with the permission of BMJ. (**C**) Bilateral fundus-based shared ResNet model predicting CVD risk, eGFR (renal function), systolic blood pressure, and diabetic retinopathy, taken from [123] with the permission of Elsevier

From coronary artery calcium to clinical decision support, multimodal frameworks emerged. Vaghefi et al. developed a deep-learning approach to detect elevated 10-year ASCVD (see Fig. 6A) risk using retinal images (plus basic demographics) with external validation [121]. Event prediction was then tightened in real-world terms. Qian et al. evaluated 5-year MACE prediction from UK Biobank retinal images [122], as shown **in** Fig. 6B, and Mellor et al. tested whether adding a deep-learning retinal score marginally improves CVD prediction in people with diabetes (see Fig. 6C), connecting retinal AI to incremental value over established clinical risk factors [123]. Finally, modality expansion links atherosclerosis to microvascular dysfunction. OCTA studies report lower retinal microvascular density and perfusion in CAD or coronary microvascular dysfunction contexts (e.g., CAD severity associations and revascularization cohorts), providing a mechanistic bridge from plaque burden to downstream microcirculatory impairment [124, 125].

### Diabetes and systemic microvascular complications prediction

Diabetes is commonly described as a “blood sugar disease,” but it is even more accurately a microvascular disease involving the whole body. Hyperglycemia, oxidative stress, and inflammation are the mechanisms that lead to microvascular damage to the retina, kidney, and peripheral nerves. The retina is also very amenable to examination, making retinal imaging a feasible and repeatable technique for evaluating microvascular disease throughout the body. The role of artificial intelligence not only involves increasing the accuracy of diagnosis of diabetic retinopathy (DR) but also the role of retinal images in the management of diabetes.

This continuum begins with the detection of DR as proof of utility. Gulshan et al. showed that deep learning is capable of grading referable DR from fundus photographs, thus proving that the disease signal is strong in retinal images [126]. Ting et al. expanded the paradigm with multiethnic training/validation and detection of related eye diseases, tackling generalizability early [127]. The next logical step was workflow realism. Abràmoff et al. demonstrated an autonomous AI system for DR detection in primary care settings, marking a move from algorithms to deployable pathways [128]. Prospective testing in India then reinforced that these systems can generalize in real screening programs [129].

**Fig. 7:**
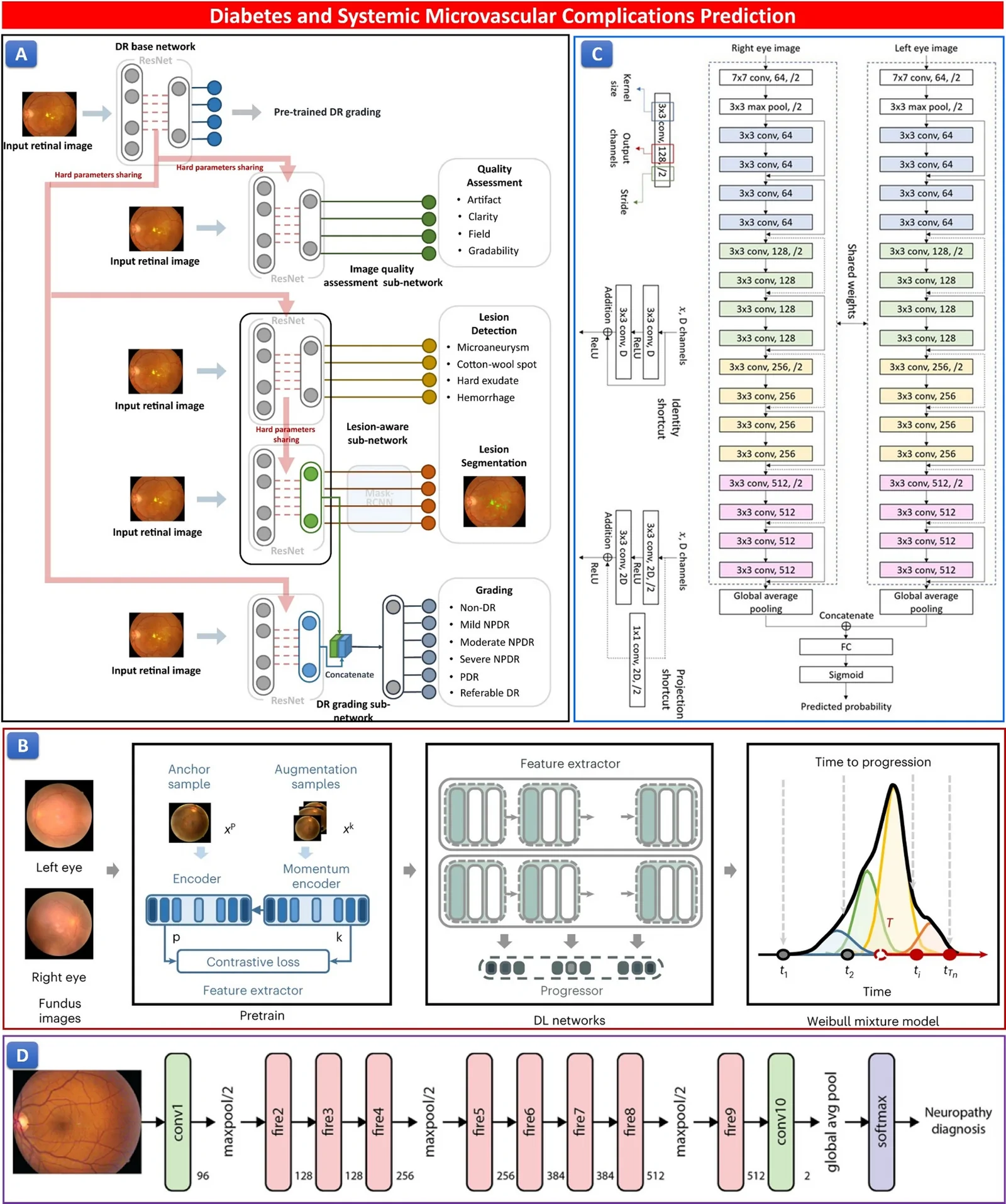
(**A**) Multi-task retinal deep learning model predicting diabetic retinopathy grade, lesion detection/segmentation, and image quality, enabling assessment of systemic microvascular damage, replicated from [130] with the permission of Springer. (**B**) Contrastive pretraining and survival modeling framework predicting time to progression of diabetic complications from bilateral fundus images, taken from [131] with the permission of Springer. (**C**) Shared-weight bilateral CNN predicting probability of DR and diabetes-related microvascular risk from right and left eye images, taken from [132] with the permission of Oxford. (**D**) Deep CNN architecture for automated diabetic neuropathy diagnosis using retinal fundus images, taken from [133] with the permission of MDPI

At the population scale, DeepDR used large real-world screening data to operationalize multitask pipelines (quality control, lesions, grading), consolidating DR AI into an end-to-end screening tool, as shown in Fig. 7A [130]. From here, the field naturally moved from “detect DR now” to “predict what happens next”: DeepDR Plus predicts time-to-progression using fundus images (see Fig. 7B), enabling personalized screening intervals, an important bridge from diagnosis to longitudinal management [131].

Once AI proved it could read subtle, preclinical signals, researchers asked whether the same images can identify systemic diabetic state itself: a UK Biobank model demonstrated screening for type 2 diabetes from retinal photos [134], and Zhang et al. showed detection (and incidence prediction) of both type 2 diabetes and chronic kidney disease (CKD) from fundus images hinting that retina captures shared microvascular/metabolic signatures [135].

That directly connects to systemic complications. Sabanayagam et al. developed deep learning to detect CKD from retinal images [136], while Kang et al. targeted early renal function impairment progressively tightening the link from generic risk to actionable kidney screening [137]. In diabetes-specific renal disease, Betzler et al. predicted diabetic kidney disease (DKD) from retinal photographs (as shown in Fig. 7C), and DeepDKD went further by aiming to detect DKD and even differentiate isolated diabetic nephropathy from non-diabetic kidney disease positioning retinal AI as a potential “non-invasive biopsy” adjunct for triage [132, 138]. Finally, the systemic narrative is extended to kidney: Cervera et al. investigated whether deep learning on fundus images could detect diabetic peripheral neuropathy (as seen in Fig. 7D), proposing that common microvascular/neural damage could be observed in the eye [133].

Throughout Sect. 5.1 to 5.4, there is a clear gradient of maturity in the evidence base. Retinal AI for diabetic retinopathy screening is the most ready for implementation (technology readiness level, TRL ≈ 5–7), as it has already proven its worth in real-world settings, including key prospective trials of autonomous systems in primary care. In contrast, applications focusing on hypertension/blood pressure monitoring, stroke risk, and coronary artery disease/atherosclerosis are still largely at TRL ≈ 3–5. These have enormous potential but are largely based on retrospective development and external validation studies, whose results are liable to biases due to device/site transfer or clinical factors such as treatment and comorbidities. Stroke is the most developed potential next application, as new foundation models specifically learn eye-brain associations and utilize intermediate phenotypes (such as silent brain infarction) to improve the risk of both incident and recurrent stroke, thus achieving TRL ≈ 5–6 in some initiatives. The future direction is consistent across all subsections: prospective multicenter trials integrated into real-world care pathways, assessment of uncertainty and fairness, provision of strong domain generalization capabilities across a wide range of imaging hardware and populations, and development of multimodal fusion (fundus imaging plus OCT/OCTA and underlying basic clinical variables) with longitudinal outcome assessment to prove overall benefit over existing risk prediction models. This approach casts retinal AI as an opportunistic triage and surveillance tool rather than a replacement for conventional diagnostic tools (Table 3).

**Table 3:** Comparative summary of representative AI-enabled retinal case studies across Sect. 5.1–5.4

Ref.	Representative AI case study	Input & scale	Model/approach (how it helps)	Key performance (and why it matters)	What improved because of AI	Indicative TRL*
[100]	Poplin et al., 2018	Color fundus photos (UK Biobank + EyePACS)	CNN multitask prediction of CV risk factors	SBP MAE ≈ 11.23 mmHg *(error for BP estimation; lower is better)*; MACE AUC ≈ 0.70 *(discrimination; higher is better)*.	Turns a routine eye photo into “opportunistic” BP/CV risk estimation (no extra test).	4–5
[114]	DeepRETStroke (2025)	Retinal photos; large-scale external validation	Foundation-model pretraining + downstream stroke tasks	Incident stroke AUC 0.901 (internal), 0.728–0.895 (external); Recurrent stroke AUC 0.769; Silent brain infarction AUC 0.797 *(risk discrimination)*.	Retina becomes a low-cost “neurovascular sensor” for silent disease + future stroke risk.	5–6
[139]	Govindaiah et al. (UK Biobank report, 2025)	Retinal images + socio-demographic/clinical features	ML risk model for future stroke	5-yr incident stroke AUC 0.83, 10-yr AUC 0.79; outperforming Framingham (0.73)/CHADS2 (0.74) *(comparative discrimination)*.	Better long-horizon risk stratification than classical scores using retinal data.	4–5
[119]	Rim et al., RetiCAC (2021)	216,152 retinal photos (SK, Singapore, UK)	DL-predicted CAC proxy + Cox risk stratification	CAC presence AUC 0.742 *(subclinical atherosclerosis detection)*; prognostic c-index 0.71 *(time-to-event ranking)*; improved reclassification over PCE.	Non-CT pathway toward CAC-like risk information—valuable in low-resource settings.	4–5
[105]	Lee et al., 2023 (Hypertension cohort)	23,148 hypertensive patients; retina + routine clinical data	Multimodal learning (retina-only vs. retina+clinical) for MACE	AUROC 0.781 (retina-only) → 0.872 (retina + clinical) *(incremental predictive utility)*.	Clear gain when fusing retinal phenotype with routine clinic variables.	5–6
[126]	Gulshan et al., 2016	Fundus photos; multi-site validation	CNN for referable DR detection	AUC ≈ 0.99; sensitivity ~ 97%, specificity ~ 93–94% *(screening operating point)*.	Enables scalable DR triage with high sensitivity (fewer missed referables).	4–5
[130]	DeepDR, 2021	466,247 images training + 3 external test sets	Multi-task DL (quality + lesion detection + DR grading)	DR grade AUCs: mild 0.943, moderate 0.955, severe 0.960, PDR 0.972; external 0.916–0.970 *(full-spectrum grading)*.	Moves beyond “referable/non-referable” into early→late grading + QC.	6–7
[136]	Sabanayagam et al., 2020 (CKD)	SEED + external SP2 & BES cohorts	Image DLA + “hybrid” (image + risk factors)	SEED validation: AUC 0.911 (image), 0.938 (hybrid); external AUCs 0.733–0.858 *(screening feasibility + transportability)*.	Adds kidney-risk screening to eye programs without blood/urine first-line.	4–5

## AI-derived retinal biomarkers for neurodegenerative disorders

### Alzheimer’s disease prediction

Alzheimer’s disease (AD) is usually diagnosed late because today’s most trusted biomarkers (PET, CSF, advanced MRI) are expensive, invasive, or not widely available. The retina offers a practical “window” into brain-linked biology because it shares neural tissue and a microvascular network that can reflect neurodegeneration, vascular dysfunction, and inflammation. The key idea behind retinal AI for AD is not that clinicians can “see Alzheimer’s in the eye” reliably by inspection, but that subtle, high-dimensional patterns in color fundus photos, OCT, and OCTA often below human perception can be learned by algorithms and used for screening, risk stratification, and trial enrichment (i.e., finding people who should be prioritized for confirmatory brain biomarkers).

**Fig. 8:**
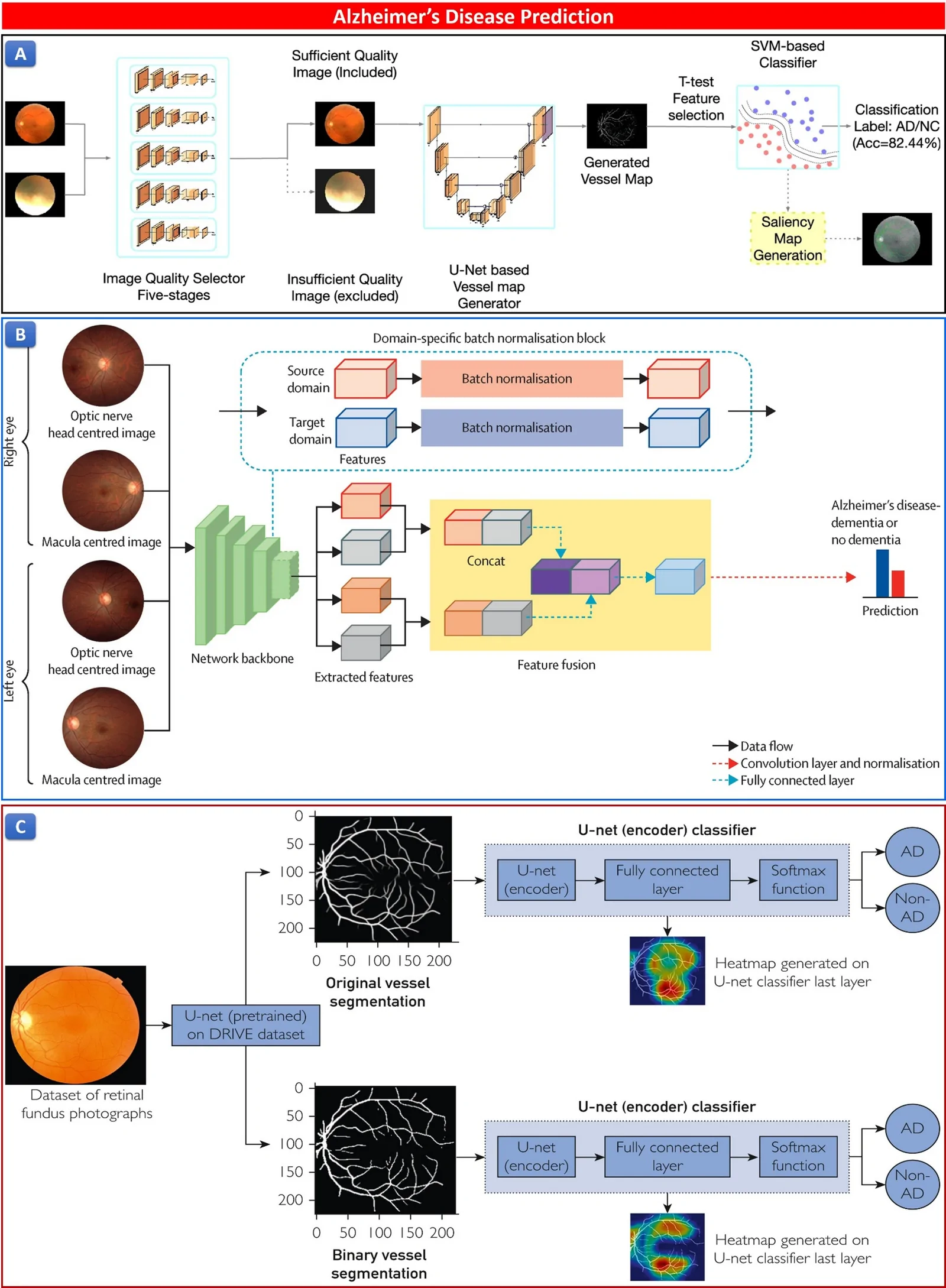
(**A**) Vessel-segmentation + SVM framework using retinal images to classify Alzheimer’s disease vs. normal control, taken from [140] with the permission of Springer. (**B**) Domain-adaptive deep learning model fusing bilateral optic nerve and macula-centered images to predict Alzheimer’s disease/dementia status, taken from [141] with the permission of Elsevier. (**C**) U-Net–based vessel segmentation and encoder classifier predicting AD vs. non-AD, with heatmap-based interpretability, taken from [142] with the permission of Elsevier

Research has progressed from feature-based vessel analytics to end-to-end and multimodal deep learning. Early work used modular ML on retinal vasculature features to classify AD (see Fig. 8A), showing that vessel geometry can carry discriminative signal [140]. A significant milestone towards practical screening was the multi-center trial involving training an EfficientNet-based bilateral model on four retinal images per individual with domain adaptation on 11 datasets, which achieved internal AUROC ~ 0.93 on AD-dementia classification and AUROCs on testing for AD diagnoses ranging ~ 0.73–0.91, including assessment based on amyloid PET status in subsets, as shown in Fig. 8B [141]. More recently, self-supervised learning has been employed to mitigate the need for large, fully labeled AD datasets. Dumitrascu et al. described a self-supervised CNN of the BERT type (pre-trained on ~ 178k UK Biobank fundus images) that performed better than a U-Net vessel segmentation pipeline, with very high accuracy in their UK Biobank and in-house evaluations (Fig. 8C), and where attention maps highlighted small vascular branches [142]. In parallel, studies have broadened the target from “diagnose AD” to “measure cognition”.

Corbin & Lesage used EfficientNet with fundus images plus metadata to predict cognitive scores, supporting the idea that retinal AI can provide continuous, preclinical proxies rather than only case/control labels [143]. Multimodal learning is another recurring theme that adding multiple signals can help improve robustness. Shi et al. trained models on fundus (macula+disc fields) + OCT data, and found that the multimodal model performed best for screening cognitive impairment (AUC ~ 0.82 internal, ~ 0.78 on two external validations), with heatmaps indicating optic disc areas in fundus images and macular/optic disc areas of the retina-choroid in OCT images [144]. On the OCTA side, deep learning is increasingly used as a “measurement engine” to standardize microvasculature quantification. Xie et al. used deep segmentation to extract layer-specific OCTA vessel metrics and FAZ geometry, then related these biomarkers to AD/MCI status (e.g., reduced vessel densities/bifurcations in specific vascular plexuses) [145]. Moving beyond classic CNNs, Eye-AD introduced a graph-based approach that models intra- and inter-layer relationships in OCTA; across 5751 OCTA images from 1671 participants, it reported strong internal/external AUCs for EOAD (~ 0.94/0.90) and MCI (~ 0.86/0.80) [146]. Experimental modalities are also entering the AI pipeline. A memory-clinic hyperspectral retinal imaging study applied ML against amyloid-related labels, suggesting a path to “biochemistry-aware” retinal screening (though still early-stage) [147].

Finally, longitudinal “oculomics” is emerging: a deep-learning retinal aging biomarker (RetiPhenoAge) was associated with future cognitive decline and incident dementia, with replication in UK Biobank an important shift from cross-sectional classification to risk forecasting [148].

### Parkinson’s disease prediction

Parkinson’s disease (PD) is still diagnosed mainly from clinical signs, and by the time clear motor symptoms appear, substantial neurodegeneration has often already occurred. This is why “accessible early biomarkers” are a major unmet need, especially ones that can be deployed at scale. The retina is attractive because it is neural tissue with a dense microvascular network, and it can be imaged quickly (fundus) or layer-by-layer (OCT/OCTA) in routine eye clinics. The new paradigm is from “can we see PD in the eye?” to “can AI learn subtle retinal signatures, structural, vascular, and functional, that track risk, severity, and progression?” That is, retinal AI is being framed as a front-door screening/triage and monitoring device: low-cost, non-invasive, and possibly able to identify patients who should be referred for confirmatory neurologic evaluation.

**Fig. 9:**
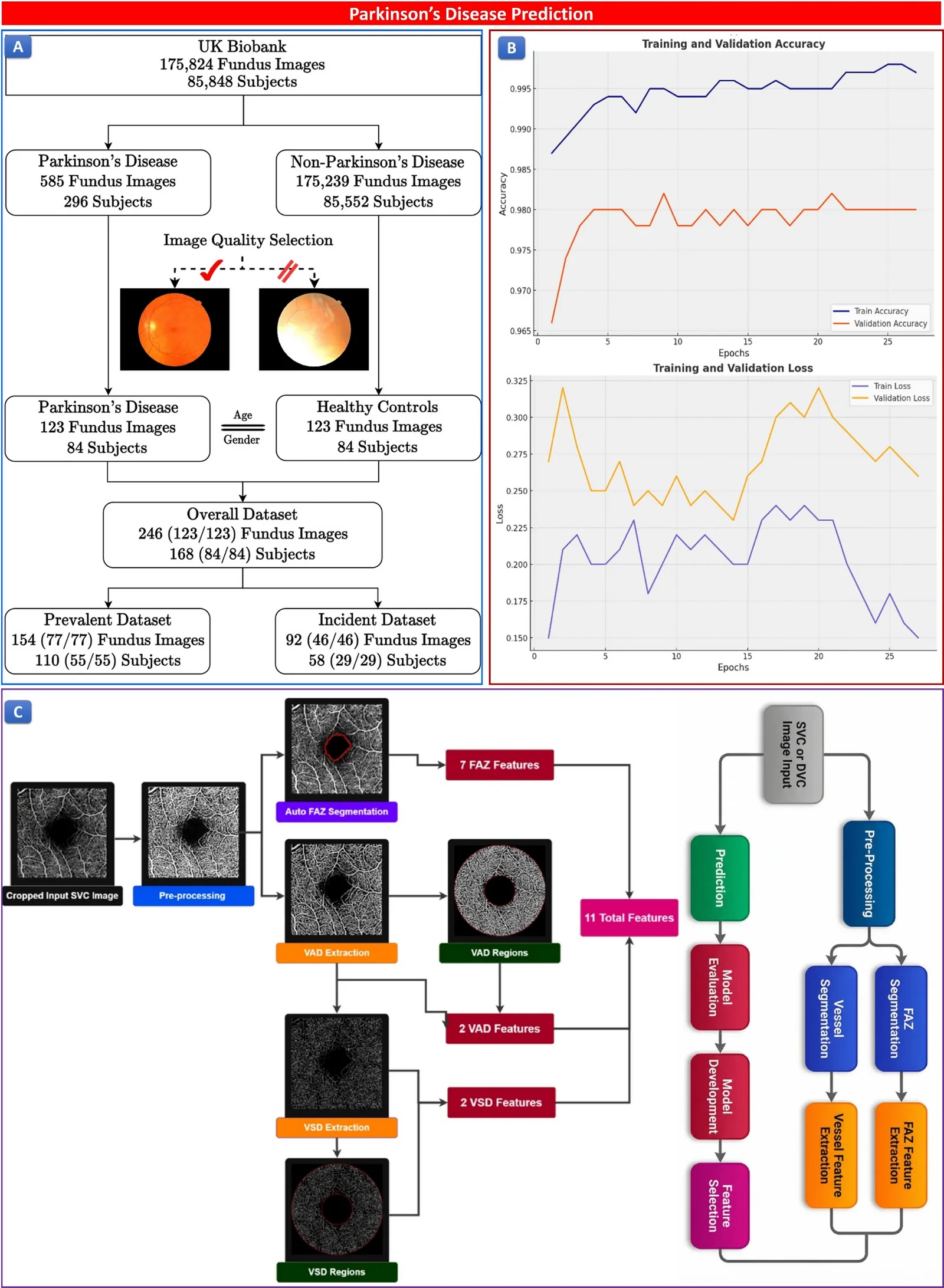
(**A)** UK Biobank–based fundus image framework predicting PD vs. healthy controls, including prevalent and incident PD classification, taken from [150] with the permission of Springer. (**B**) Deep learning training/validation performance analysis for PD detection from retinal images (accuracy and loss curves), taken from [152] with the permission of Elsevier. (**C**) Retinal microvascular feature extraction pipeline (FAZ, VAD, VSD features) to predict PD risk from OCTA/fundus images, taken from [153] with the permission of Springer

Much of the current evidence has been generated by large cohorts that have first identified what matters in change and then developed AI to identify it. For instance, in a Neurology study using AlzEye (154,830 patients) and UK Biobank, thinner inner retinal layers on OCT, particularly GCIPL and INL, were found to be associated with prevalent PD and future incident PD (although this was small), suggesting the signal can precede diagnosis [149]. A complementary longitudinal PD study examined OCT over time and found accelerated pfGCIPL thinning in PD, thinning patterns associated with cognitive decline, and validated components using AlzEye, shifting the story from “detection” to “progression biology” [61]. On the modeling side, Tran et al. trained deep networks on UK Biobank fundus photographs and reported that PD can be separated from matched controls with ~ 68% accuracy and that models can predict incident PD ahead of diagnosis across multi-year windows, supported by attribution maps for interpretability (see Fig. 9A) [150]. Rather than diagnosing PD, Ahn et al. used fundus+deep learning to estimate neurologic dysfunction severity (H&Y and UPDRS-III), hinting that retinal AI may become a “digital severity marker,” not just a classifier [151].

Another strategy reframes the retina as a biological aging readout. A deep-learning “retinal age gap” was independently associated with incident PD risk (HR ~ 1.10 per 1-year gap), with 5-year risk prediction comparable to established risk factors [154]. Multimodal architectures then attempt to stabilize performance by fusing complementary inputs, Richardson et al. combined OCT GC-IPL maps, OCTA macular images, and ultra-widefield fundus/autofluorescence in a CNN framework, reflecting the intuition that PD signatures are not purely vascular or purely neuronal [155]. At the “clinical-AI bridge,” Ganji et al. integrated OCT biomarkers with clinical features in an explainable pipeline (SHAP/LIME) and reported high diagnostic discrimination (see Fig. 9B), illustrating how retinal measurements can be made more actionable when tied to symptoms and exam findings [152]. For OCTA, ensemble ML has been explored to classify PD using microvascular metrics, suggesting a route for AI to standardize OCTA-derived biomarkers across noisy measurements, as depicted in Fig. 9C [153]. Finally, Broader “neurodegenerative fundus” models such as FundusNet explicitly include PD as a target, supporting the idea of one retinal backbone model adapted to multiple neurologic conditions [156].

### Multiple sclerosis and other neuroinflammatory diseases prediction

Multiple sclerosis (MS) and associated neuroinflammatory conditions (e.g., optic neuritis, NMOSD, MOGAD) are characterized by inflammation-mediated damage to myelin and axons, but traditional monitoring is still heavily dependent on clinical relapses, disability scales, and MRI technology that can be expensive, time-consuming, and not always sensitive to subtle disease progression. Retinal imaging provides a complementary “CNS-outpost” biomarker. OCT measures layer-specific neuroaxonal injury (RNFL, GCIPL), and OCTA provides a microvascular component potentially informative of neurovascular and hypoxic pathophysiology. The application of AI in this area is functional and translational, it takes complex, noisy retinal images and translates them into standardized, reliable data and predictive models, allowing for earlier diagnosis of subclinical injury, improved phenotyping (inflammatory vs. neurodegenerative vs. vascular predominant), and potentially scalable monitoring in clinical practice.

**Fig. 10:**
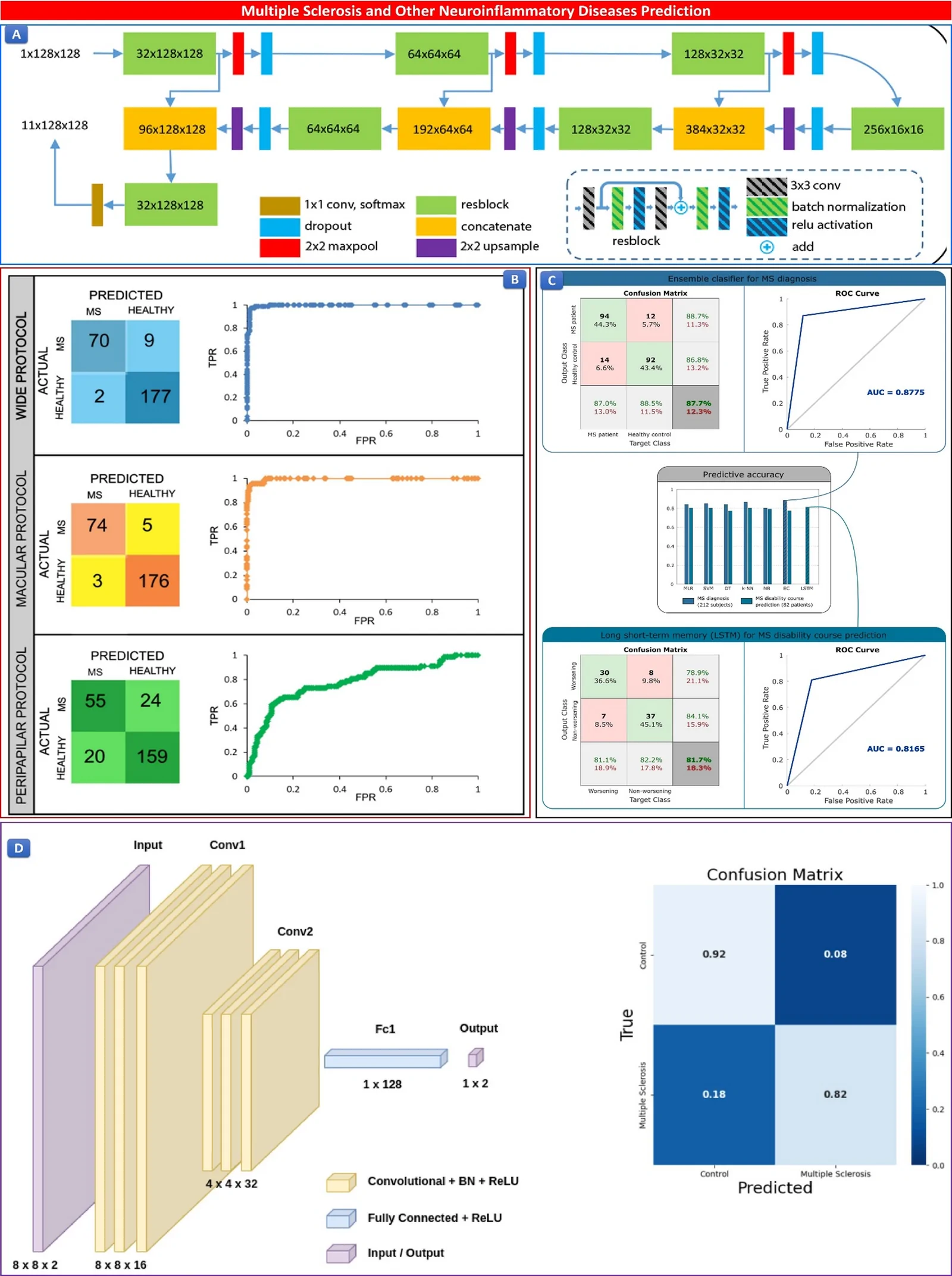
(**A**) Deep residual U-Net–based architecture predicting MS vs. healthy controls from retinal images, taken from [157] with the permission of Optica. (**B**) Performance evaluation across widefield, macular, and peripapillary protocols for MS classification, using confusion matrices and ROC curves, taken from [159] with the permission of PLOS. (**C**) Ensemble and LSTM-based models predicting MS diagnosis and disability progression (worsening vs. non-worsening) with ROC/AUC analysis, taken from [160] with the permission of Elsevier. (**D**) CNN classifier for automated multiple sclerosis detection, validated using confusion matrix performance metrics, taken from [163] with the permission of Elsevier

AI research in MS has been a logical progression. Automate “what to measure,” then learn “what it means.” Deep learning layer segmentation, first shown in MS OCT with topology-informed surfaces and boundary modeling (see Fig. 10A), improved thickness maps’ scan and user variability, a prerequisite for downstream biomarkers [157, 158]. With these measurements, initial ML research indicated that OCT-derived structural information could aid in earlier MS diagnosis, such as swept-source OCT + ML for early detection and multi-feature classifiers correlating retinal thinning to disease, as shown in Fig. 10B [159]. The next phase was clinically informed modeling. Montolío et al. integrated OCT with clinical data for MS diagnosis and disability course prediction (including sequence models for longitudinal outcomes, see Fig. 10C), moving AI research from “screening” to “forecasting” [160]. The following phase highlighted trustworthy decision support. For example, Dongil-Moreno et al. applied XAI to OCT zone features and inter-eye asymmetry for early RRMS diagnosis (reported sensitivity ~ 0.86 and specificity ~ 0.90), while Hernandez et al. concentrated on explainability as a necessary step for clinical acceptance [161, 162].

Concurrently, “image-first” pipelines explored the generalization of results across centers: Ortiz et al. posited OCT + AI as a biomarker-driven early diagnostic strategy (see Fig. 10D) [163], and Khodabandeh et al. compared traditional models across two centers to demonstrate the utility of discrimination even without extensive deep learning datasets [164]. Beyond OCT, the scope of retinal biomarkers is expanding. Autoencoder-based feature learning on IR scanning-laser ophthalmoscopy (IR-SLO) imaging proposes the existence of different vascular/morphological markers for MS diagnosis, and specific models have been developed to identify previous optic neuritis based on particular OCT scan patterns, which are important given the strong influence of ON history on retinal morphology [165]. Lastly, “other neuroinflammatory diseases” are making their way through differential diagnosis and pediatric stringency. OCTA/OCT patterns have been used to differentiate MS from NMOSD, and ML classifiers in pediatric patients have attempted to differentiate MS from other demyelinating disorders based on OCT patterns more representative of the diagnostic gray zone than case-control studies [166, 167].

### Cognitive decline and brain aging prediction

Cognitive decline and “brain aging” are trajectories, not diagnoses, that are fueled by vascular risk, neurodegeneration, inflammation, and reserve. This introduces a practical screening paradox: MRI/PET/CSF (and even blood biomarkers) are not always feasible on a population level, whereas cognitive tests may fail to detect early, subclinical changes. Retinal imaging is appealing because it is rapid, repeatable, and reflects both neuronal tissue and microvasculature. AI’s role is to translate subtle retinal features into quantitative, longitudinal markers either as continuous “aging” indices or as proxies for brain health (such as small vessel disease burden) that lie upstream of dementia and cognitive impairment.

**Fig. 11:**
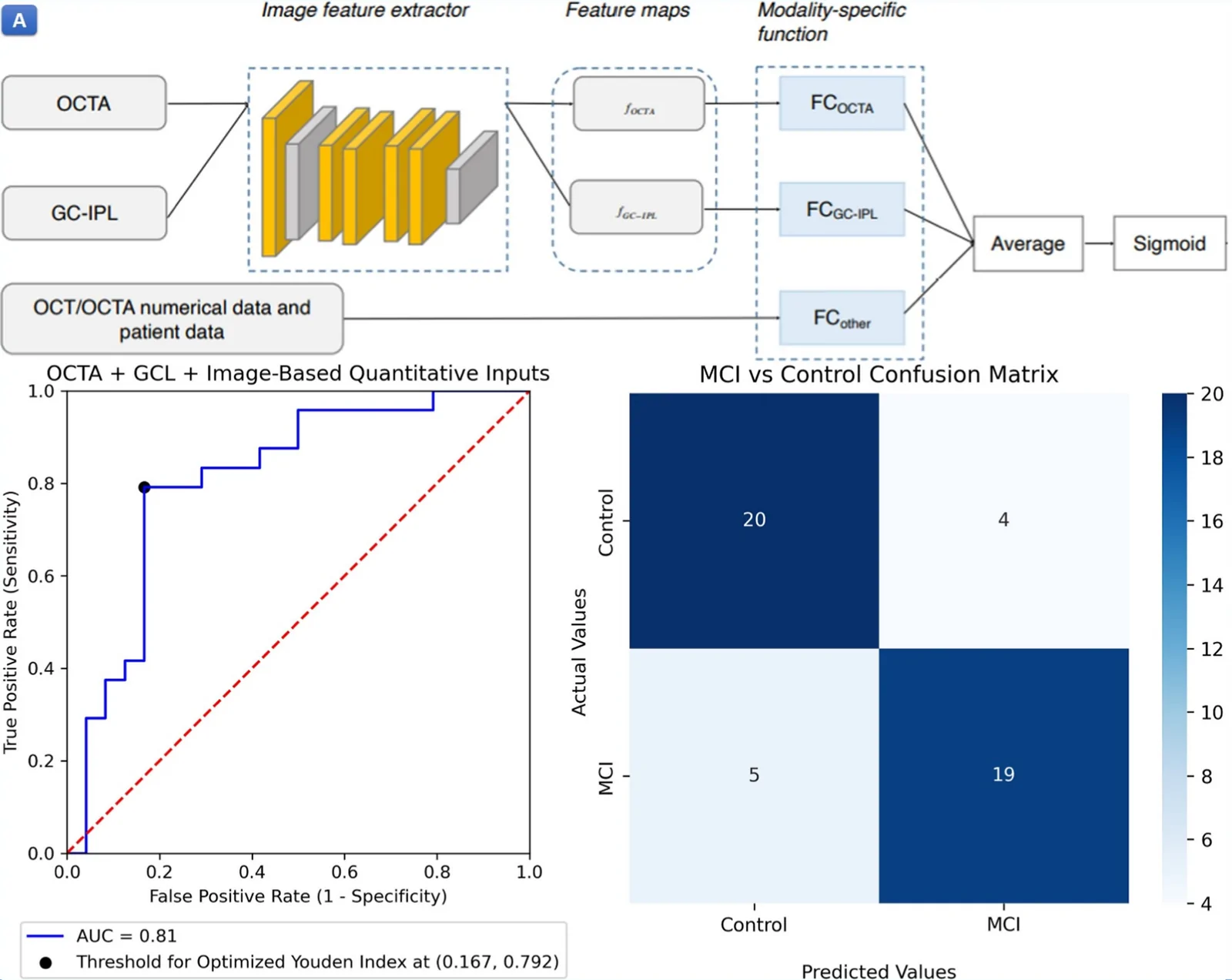
Multimodal deep learning framework combining OCTA, GC-IPL, and clinical data to predict mild cognitive impairment vs. healthy controls, enabling early detection of cognitive decline and brain aging, taken from [173] with the permission of Elsevier

One way to integrate the evidence is to think of it as a pipeline from generic aging → microvascular injury → cognitive phenotype. On the aging side, deep learning has been applied to predict biological age from fundus photographs (Nusinovici et al.), positing that the retina encodes a representation of systemic aging independent of chronological age [168]. Similar “retinal age gap” models (predicted age - actual age) offer a reusable template for brain aging studies because they distill complex images into a single, interpretable measure of deviation from expected [169]. Turning from aging to mechanism, AI-assisted retinal vasculometry platforms (such as QUARTZ) enable automatic vessel segmentation and measurement of width and tortuosity at scale; in UK Biobank, AI-assessed vasculometry measures demonstrated graded relationships with composite cognition scores across > 63,000 individuals, implying an “microvascular cognition” measure within reach [170, 171]. The next link is to brain imaging proxies. Cho et al. demonstrated that deep learning on fundus photographs can partially reconstruct MRI white matter hyperintensity (WMH) burden (a marker of cerebral small-vessel disease strongly associated with cognitive impairment), with models centered on macula and vasculature [172]. Finally, orthogonal work applying OCT/OCTA to CNNs has also discriminated normal cognition from MCI (see Fig. 11), implying a “neurostructural + vascular” strategy for brain health screening [173].

In short, in 6.1–6.4, the state of retinal AI for neurodegeneration and brain health is clearly very promising but also very much in the pre-deployment phase. The majority of AD, PD, MS/neuroinflammation, and brain-aging/cognitive-decline work is at TRL ~ 2–4, since these are largely retrospective, case-control, or enriched cohorts, and based on imperfect clinical labels rather than biomarker-validated truth on the ground. The most successful “next-step” work is that which has multi-cohort external validation and some grounding in brain truth (PET/CSF/MRI), pushing some of the AD and cognitive-aging work towards TRL ~ 4–5, and PD and MS towards TRL ~ 2–4 with high biological plausibility but less prospective workflow studies. The most “deployable” building blocks at present are not final disease models per se but the measurement and harmonization layers, high-quality OCT/OCTA segmentation, quality control, and domain adaptation which make retinal biomarkers comparable across instruments and sites and allow for longitudinal follow-up. The path forward is the same for all four sections: conduct prospective multi-site studies under real-world clinic acquisition conditions; include brain truth anchors (at least in subsets) to decrease label noise; address confounders (age, vascular disease, diabetes, ocular comorbidities, treatment); and output results in clinically actionable trajectories (risk now + expected progression + uncertainty). In this context, the most plausible near-term role for retinal AI is as a front-door triage and trial enrichment tool flagging patients for definitive biomarker testing, and enabling scalable longitudinal monitoring, while multimodal fusion (fundus + OCT + OCTA + metadata) and foundation model pretraining can help improve robustness and label efficiency along the way to higher TRLs.

### Proposed clinical workflow for real-world implementation of AI-enabled retinal imaging

The process of taking multimodal imaging using an AI-powered system from laboratory research to clinical application will have to be anchored by the design of a well-articulated process for screening, referral, and follow-up. This could start at the level of primary care clinics, diabetes or hypertension clinics, ophthalmic screening centers, memory centers in neurology or even mobile health camps. At such clinics, retinal imaging can be adopted as a noninvasive tool that is applied during screening to help determine which patients need further testing or evaluation.

**Fig. 12:**
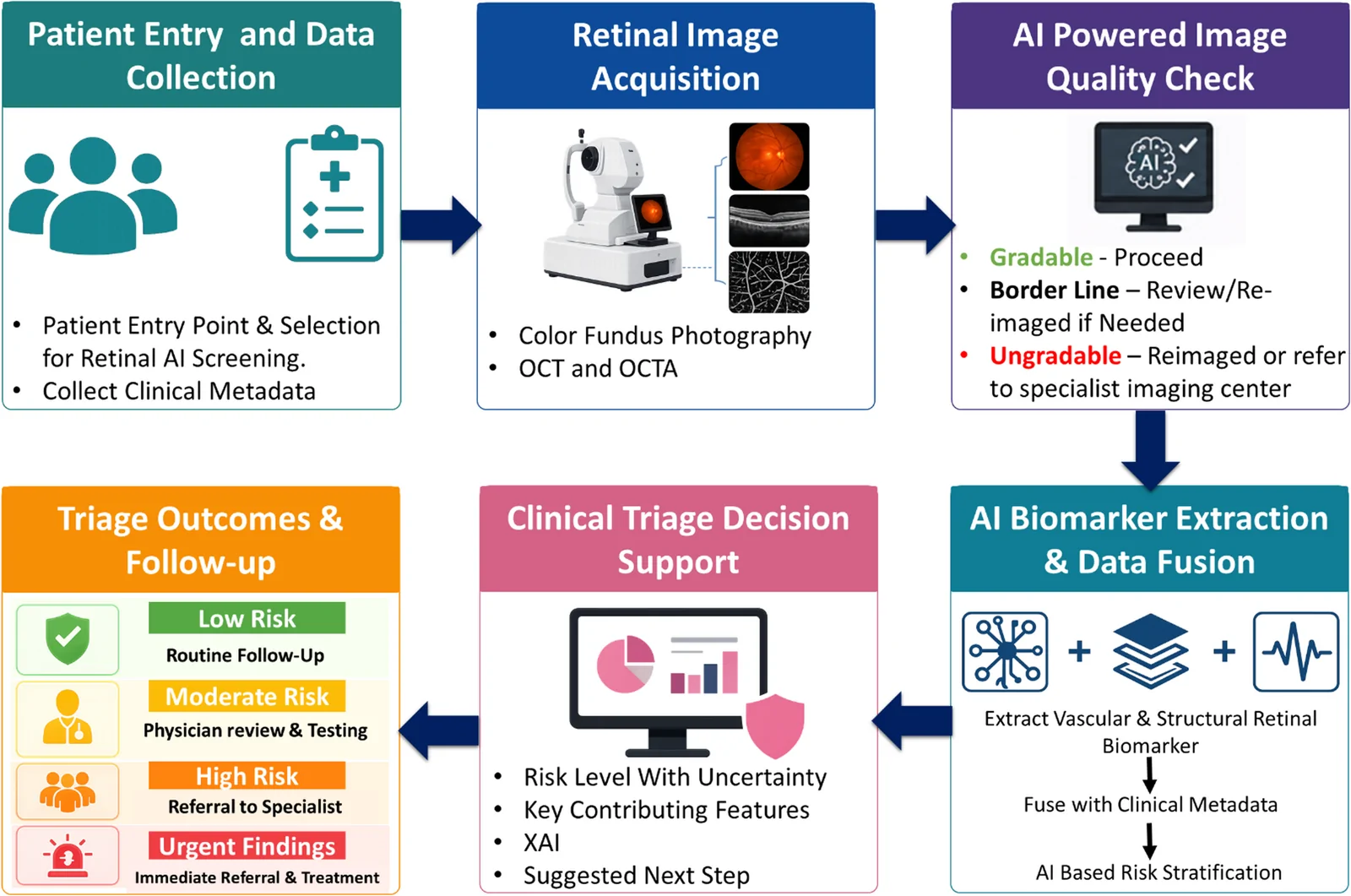
Proposed clinical workflow for real-world implementation of AI-enabled multimodal retinal imaging for systemic vascular and neurodegenerative risk stratification

The suggested workflow (see Fig. 12) starts with the process of selecting patients who need to undergo risk assessment. Patients who have certain risk factors such as diabetes, high blood pressure, chronic kidney disease, dyslipidemia, previous diseases of the cardiovascular system, signs of cognition problems, a positive family history of neurodegenerative disease, or are simply old can be chosen to undergo retinal screening. Basic data on patients that include their age, gender, blood pressure, glycemic level, lipid values, smoking, medications, ocular conditions, and neurological/vascular symptoms need to be gathered in addition to retinal images.

Following the image acquisition stage, it would involve acquiring an image by means of non-mydriatic color fundus photography as a bare minimum of the imaging modality, together with OCT and OCTA if possible. In community settings, the fundus imaging could be used as a baseline imaging modality whereas OCT/OCTA could be reserved for high-risk patients or specialist imaging centers. The very next step following image acquisition would involve an automatic AI-driven image quality check and classification into gradable, borderline, and ungradable. An ungradable image will lead to prompt re-imaging or refer to a specialist imaging center to avoid misinterpreting poor quality input data.

The following step would involve automatic extraction and multimodal fusion of retinal biomarkers. This could entail recognition of vascular biomarkers like vessel size, tortuosity, arteriovenous ratio, hemorrhage, exudates, microaneurysms, capillary density, and perfusion loss, as well as structural biomarkers like RNFL thickness, ganglion cell complex thickness, macular thickness, and neurovascular parameters derived from OCT/OCTA. These biomarkers, along with clinical metadata, could form the basis of risk outputs specific to one disease or several diseases, like cardiovascular risk, stroke risk, risk of diabetic complications, cognitive decline risk, or neurodegenerative disease risk.

The AI result must not be considered as an independent diagnosis. It must be presented as a decision-support tool for clinicians, which would comprise the patient’s risk level, the degree of uncertainty, important features that contributed to the risk score, explanations, and suggestions. The triaging system might look like this: low risk – regular follow-up and appropriate risk factor management; moderate risk – physician analysis, further imaging, or clinical testing; high risk – consultation with the relevant medical specialist (ophthalmology, cardiology, neurology, nephrology, or endocrinology); urgent ocular/systemic findings – immediate referral for further investigation and treatment.

For instance, a patient at a high risk for retinal AI stroke could be subjected to blood pressure verification, ambulatory blood pressure monitoring, risk assessment for vascular disease, and neuroimaging when appropriate. Patients who have been identified as having retinal characteristics that indicate neurodegeneration risk can be considered for cognitive screening, neurological assessment, or biomarker verification. Again, patients at risk for retinal characteristics associated with diabetic microvascular disease can be screened for nephropathy, neuropathy, or metabolic issues. Therefore, the use of retinal AI should be interpreted as one of triage, not as an alternative to current diagnostic approaches.

Finally, the application of such a system in practice necessitates a feedback and governance cycle. Information about treatment outcomes, confirmation of diagnoses, referrals, and follow-up data from patients needs to be incorporated into the process for evaluation, calibration, checking biases, and updating the algorithm. Seamless integration with electronic medical records, uniform data reporting, data privacy considerations, and human oversight will play a crucial role in its safe implementation. Through this approach, AI-assisted retinal imaging can facilitate large-scale, easy-to-use, and longitudinal screening initiatives. 

## Technical and clinical challenges

### Multicenter validation gaps + domain shift (devices/sites/populations)

Because models are trained on one camera/OCT vendor, geography, or acquisition protocol, they tend to perform poorly when moved to other settings due to differences in lighting, resolution, artifacts, population demographics, disease prevalence, and referral patterns. This is the biggest reason why “great AUROC in a paper” doesn’t work in practice: the model is learning shortcuts that are specific to the training distribution. To bridge this gap, validation in independent centers is necessary, along with domain-robust training [174, 175].

### Small annotated datasets + label noise (especially for systemic/neuro outcomes)

In many cases, labels for systemic endpoints (future stroke, amyloid-PET status, CKD progression) are sparse, costly, or noisy (ICD codes, incomplete follow-up). Small datasets drive models towards overfitting and unstable behavior, and label noise increases uncertainty and can lead to spurious correlations. Workarounds include self-supervised pre-training, weak supervision, endpoint definition, and more comprehensive longitudinal labeling (time-to-event instead of binary) [176, 177].

### Lack of prospective trials (clinical utility is not yet proven)

The Retinal-AI systems could be correct retrospectively but still not improve outcomes because they do not alter the behavior of the clinicians, referrals, compliance, and treatment choices. Prospective studies are required to demonstrate “net benefit” in terms of earlier detection, missed high-risk patients, resource allocation (who really needs MRI/PET), and safety (no harmful false reassurance). This is the critical transition from TRL ~ 3–5 to TRL ~ 6–8 [178–180].

### Multimodal fusion reality: missing modalities, misalignment, and data timing

In actual clinics, not all patients will have fundus + OCT + OCTA; scans can be taken on different days; and systemic labs/wearables can be few and asynchronous. Fusion models with “all modalities always present” assumptions can fail. High-quality multimodal models must address missing modalities (gating/MoE), temporal alignment (time-aware models), and have proper fallbacks (minimum viable inputs with uncertainty) [181, 182].

### Calibration, uncertainty, and safety (risk scores must be actionable)

High AUROC does not imply safe behavior. A model needs to be well-calibrated (predicted risk = observed risk), offer uncertainty estimates, and enable safe thresholds (rule-in, rule-out, and “needs repeat imaging”). Otherwise, you’ll have unstable referral volumes, a lack of trust among clinicians, and medicolegal issues, particularly in screening programs [183, 184].

### Interpretability vs. performance trade-off (trust and accountability)

It is important for clinicians to understand whether the model is looking at credible areas of the retina (vessels, RNFL, non-perfusion areas) and whether the explanations are stable. However, simpler models may perform poorly, while better deep models may not be interpretable. The trade-off is “interpretable outputs around a strong model,” which includes explanation overlays, feature attribution for important biomarkers, counterfactual validation, and error audits related to failure modes [185, 186].

### Privacy, federated learning, and governance (data sharing constraints)

Multimodal precision systems need big data, but sharing health data is limited by consent, geography, and institutional policies. Federated learning and privacy-preserving analytics are useful, but they add complexity to governance: who owns the model, how is the model updated and audited for bias, and how are sites compensated and protected. Sound governance is as important as the algorithm [187, 188].

### Clinical acceptance barriers (workflow fit, accountability, and “who acts”)

Even if it is accurate, a tool will fail if it does not integrate with workflow: who orders imaging, who interprets results, who initiates confirmatory testing, and how referrals are handled. Implementation will also depend on accountability (human-in-the-loop vs. autonomous), education, and defined clinical pathways (e.g., “retinal AI high stroke risk → confirm BP/ABPM + targeted neuroimaging only for top tier”) [189, 190].

### Interoperability and standardization (EHR/DICOM/metadata quality)

Multimodal fusion requires good metadata (device type, scan quality, laterality, time stamps) and clean connectivity (PACS/EHR). Without standards, fusion pipelines will fail, and models will quietly misinterpret incorrect or incomplete context. “Hidden work” is creating consistent data pipelines, QC gates, and outputs that can be quickly understood by clinicians [191, 192].

### Health-economic proof (cost-effectiveness and scalability)

For screening/triage tools, the winning argument is economic: fewer unnecessary expensive tests, earlier prevention, and better allocation of specialist time. Without cost-effectiveness evidence, payers and hospitals hesitate, even if performance is strong. Economic modeling and real-world implementation studies must accompany algorithm validation Table 4 [193, 194].

**Table 4:** Comparative table: challenges, solutions, why they work, and a 5-year roadmap

Challenge	Possible solutions	Why it works	Next 12–24 months	Next 3–5 years
Multicenter validation + domain shift	External validation, domain adaptation, site-stratified reporting, drift monitoring	Proves robustness; prevents “single-site” overclaiming	Publish multi-center benchmarks; add drift dashboards	Routine post-market surveillance; continual learning under governance
Small labeled datasets + label noise	SSL/foundation pretraining, weak supervision, better endpoint definitions, time-to-event labels	Reduces label dependence; improves generalization	Pretrain on unlabeled retina; harmonize labels across sites	Large federated cohorts with standardized longitudinal outcomes
Lack of prospective trials	Pragmatic trials, impact studies (referrals/outcomes), safety endpoints	Moves from accuracy to real clinical value	Pilot workflow trials in screening programs	Multi-site randomized/stepped-wedge trials; guideline inclusion
Missing modalities + temporal mismatch	Modality-agnostic encoders, gating/mixture-of-experts, time-aware fusion	Works even when inputs are incomplete or asynchronous	Build “minimum viable input” + fallback logic	Mature digital-twin style longitudinal fusion models
Calibration + uncertainty	Temperature scaling, Bayesian/ensembles, conformal prediction, threshold policies	Makes outputs actionable and safer	Add calibration plots + uncertainty in reports	Standardized clinical-grade risk calibration across populations
Interpretability vs. performance	Heatmaps + concept checks, stable explanations, clinician-facing summaries	Builds trust while keeping strong models	Standardize explanation QA and failure-mode audits	Interpretable-by-design multimodal models for regulated use
Privacy, FL, governance	Federated learning, secure enclaves, data use agreements, audit trails	Enables scale without centralized data	Launch FL pilots across 3–5 sites	Federated “global retinal networks” with continuous auditing
Clinical acceptance/workflow	Clear triage pathways, human-in-loop roles, training, escalation rules	Adoption depends on who acts and when	Map workflows + referral policies; clinician co-design	Integration into routine pathways (primary care/pharmacy/telehealth)
Interoperability/standardization	DICOM/metadata standards, QC automation, EHR integration APIs	Prevents silent data errors; ensures repeatability	Implement QC gates + standardized output templates	Fully interoperable multimodal CDSS embedded in EHR/PACS
Health-economic proof	Cost-effectiveness models, resource impact studies, payer alignment	Drives scaling and reimbursement	Early economic models using pilot data	Reimbursement pathways + large-scale adoption based on ROI

### Ophthalmic confounders and imaging-specific limitations

The use of AI-assisted retinal imaging is fraught with potential pitfalls, as the retinal biomarkers are subject to several non-systemic ophthalmic confounders. Changes in OCT parameters that may constitute valid biomarkers include RNFL, GCIPL/GCC, macular thickness, thickness of outer retinal layers, and choroidal thickness. However, any of these changes are not specific to a particular disease. Hence, retinal nerve fiber layer or ganglion cell complex thinning may occur not only due to neurodegeneration but also as a result of glaucoma, optic neuritis, high myopia, aging, or retinal vascular disease.

Additionally, measurement results obtained with OCT are vulnerable to segmentation errors caused by the presence of poor signal strength, macular edema, epiretinal membrane, drusen, high myopia, or an unusual retinal contour. This may cause misinterpretation of thickness and subsequent erroneous conclusions regarding the course of a disease. OCTA biomarkers like vessel density, perfusion density, capillary dropout, and foveal avascular zone may also be distorted by numerous imaging artifacts caused by motion, blink, projection, shadowing, defocus, or slab-segmentation.

Patient-level confounders may affect AI’s performance by altering image quality, changing magnification, distorting vascular or structural measurements, or introducing systemic bias. The list includes cataracts, corneal opacity, vitreous haze, small pupil size, poor fixation, ocular media opacity, axial length, refractive error, high myopia, and local ocular diseases like diabetic retinopathy, age-related macular degeneration, retinal vein occlusion, glaucoma, or prior ocular surgeries.

Thus, in further retinal-AI research (see Table 5), one should consider incorporating image-quality control, artifact detection, segmentation validation, axial length/ocular refraction normalization, ocular comorbidities registration, standardization of devices/vendors, uncertainty estimates calculation, and human-in-the-loop review [68, 195–197].

**Table 5:** Ophthalmic confounders and imaging-specific limitations affecting AI-enabled retinal risk prediction [68, 195–197]

Confounder/Limitation	Effect on Retinal Imaging	Possible Impact on AI Model	Recommended Mitigation
Cataract	Reduces image clarity, contrast, and signal strength	False abnormal prediction or missed subtle pathology	Image quality threshold; repeat imaging after cataract management if needed
Corneal opacity/dry eye	Causes blur, scatter, and poor fixation	Reduced vessel/layer visibility; unstable model output	Lubrication, repeat scan, quality-control flag
Vitreous opacity/floaters/hemorrhage	Causes shadowing and localized signal loss	May mimic non-perfusion or lesion-like abnormalities	Manual review; artifact masking; repeat imaging
Small pupil/poor fixation	Reduces field quality and increases motion artifacts	Ungradable images or unreliable predictions	Pupil dilation if appropriate; fixation coaching; repeat imaging
High myopia/long axial length	Causes magnification error, retinal thinning, disc tilt, peripapillary atrophy	May mimic neurodegeneration or microvascular loss	Record axial length/refractive error; apply magnification correction; subgroup analysis
Glaucoma/optic neuropathy	Causes RNFL and GCIPL/GCC thinning	May be confused with neurodegenerative disease-related thinning	Include glaucoma status; exclude or stratify affected eyes
Diabetic retinopathy/macular edema	Alters vessels, macular thickness, hemorrhages, and exudates	May dominate systemic-risk features or bias multimodal models	Grade ocular disease separately; adjust for DR severity
Age-related macular degeneration	Alters outer retina, RPE, drusen, and macular architecture	May interfere with OCT-based neuroretinal biomarkers	Exclude advanced AMD or analyze separately
Epiretinal membrane/vitreomacular traction	Distorts retinal contour and layer boundaries	Causes segmentation errors and false thickness changes	Segmentation review; exclude severe traction
Retinal vein occlusion/vascular occlusion	Causes hemorrhage, edema, ischemia, and vessel remodeling	May be misread as generalized systemic vascular disease	Clinical annotation; affected-eye exclusion or stratification
OCT segmentation error	Incorrect RNFL, GCIPL, macular, or choroidal thickness measurement	False progression or incorrect risk category	Manual review; segmentation confidence maps; correction protocols
OCTA motion/blink artifact	Creates vessel duplication, breaks, or false dropout	Incorrect vessel density or perfusion metrics	Motion correction; repeat scan; artifact detection
OCTA projection artifact	Superficial vessels appear in deeper layers	False flow signal in deep retina/choriocapillaris	Projection-removal algorithms; slab-wise review
OCTA shadowing/signal attenuation	Reduced signal below opacity, hemorrhage, or thickened retina	False capillary dropout or non-perfusion	Signal-strength correction; structural OCT correlation
Device/vendor variability	Different scan protocols, resolution, and segmentation algorithms	Poor generalization across clinics	External validation; device harmonization; domain adaptation

## Conclusions and future direction

Multimodal retinal imaging using AI capabilities marks a paradigm change from late-stage disease diagnosis to early risk assessment, real-time surveillance, and large-scale preventive medicine for systemic vascular and neurodegenerative disorders. By synergistically combining structural, vascular, and metabolic patterns in the retina with sophisticated learning paradigms, the retina can be leveraged as a feasible and noninvasive biomarker platform for cardiovascular, metabolic, and brain health. While existing literature indicates great promise in multiple diseases, further clinical translation in a broader context is still needed to ensure rigorous multicenter validation, prospective outcome-driven studies, standardized data collection, and effective bias, uncertainty, and regulatory considerations. Future directions will necessitate multimodal fusion with clinical, genomic, and longitudinal health information, as well as digital health infrastructure and cost-effectiveness studies to ensure real-world applicability. In the short term, retinal AI is most likely to find applications as a scalable triage and surveillance platform that will complement, rather than substitute for, existing diagnostic capabilities. In the long term, future advances in foundation models, explainable AI, and federated learning may enable precision screening frameworks that are equitable and effective in improving early intervention, alleviating healthcare burden, and advancing preventive neurometabolic medicine worldwide.

### Method of literature search

We conducted a comprehensive literature search to identify the relevant studies on artificial intelligence–integrated multimodal retinal imaging for the detection and risk stratification of systemic vascular and neurodegenerative diseases. Peer-reviewed articles were retrieved from the following scientific databases: PubMed/MEDLINE, Web of Science, and Scopus. These databases were selected to ensure coverage of biomedical, ophthalmic, and artificial intelligence research. The search strategy combined terms related to retaining imaging, artificial intelligence, and systemic diseases. Additional keywords such as retinal and vascular biomarkers, multimodal imaging, predictive analytics, and risk stratification were also used to expand the literature search. The findings were then synthesized to highlight current advances, clinical applications, and future challenges in AI-driven retinal biomarkers for systemic disease detection.

## Data Availability

Data is included within the manuscript.

## References

[1] Mensah GA, Fuster V, Murray CJL, Roth GA (2023) Global burden of cardiovascular diseases and risks, 1990–2022. J Am Coll Cardiol 82:2350–2473. 10.1016/j.jacc.2023.11.007 38092509 PMC7615984

[2] Livingston G, Huntley J, Sommerlad A, Ames D, Ballard C, Banerjee S, Brayne C, Burns A, Cohen-Mansfield J, Cooper C, Costafreda SG, Dias A, Fox N, Gitlin LN, Howard R, Kales HC, Kivimäki M, Larson EB, Ogunniyi A, Orgeta V, Ritchie K, Rockwood K, Sampson EL, Samus Q, Schneider LS, Selbæk G, Teri L, Mukadam N (2020) Dementia prevention, intervention, and care: 2020 report of the Lancet Commission. Lancet (London England) 396:413–446. 10.1016/S0140-6736(20)30367-6 32738937 PMC7392084

[3] Rundek T, Tolea M, Ariko T, Fagerli EA, Camargo CJ (2022) Vascular cognitive impairment (VCI). Neurotherapeutics 19:68–88. 10.1007/s13311-021-01170-y 34939171 PMC9130444

[4] Cui J, Robert C, Teh CM, Jun Yi EC, Chong JR, Tan BY, Venketasubramanian N, Lai MKP, Chen C, Hilal S (2024) Interactive effect of diabetes mellitus and subclinical MRI markers of cerebrovascular disease on cognitive decline and incident dementia: a memory-clinic study. Alzheimers Res Ther 16:214. 10.1186/s13195-024-01577-7 39363381 PMC11448036

[5] Zhang J, Zhang Y, Wang J, Xia Y, Zhang J, Chen L (2024) Recent advances in Alzheimer’s disease: mechanisms, clinical trials and new drug development strategies. Signal Transduct Target Ther 9:211. 10.1038/s41392-024-01911-3 39174535 PMC11344989

[6] Kauko A, Engler D, Niiranen T, Ortega-Alonso A, Schnabel RB (2024) Increased risk of dementia differs across cardiovascular diseases and types of dementia – data from a nationwide study. J Intern Med 295:196–205. 10.1111/joim.13733 37899293

[7] Lyu F, Wu D, Wei C, Wu A (2020) Vascular cognitive impairment and dementia in type 2 diabetes mellitus: an overview. Life Sci 254:117771. 10.1016/j.lfs.2020.117771 32437791

[8] Heidt B, Siqueira WF, Eersels K, Diliën H, van Grinsven B, Fujiwara RT, Cleij TJ (2020) Point of care diagnostics in resource-limited settings: a review of the present and future of PoC in its most needed environment. Biosensors. 10.3390/bios10100133 32987809 PMC7598644

[9] Bhaiyya M, Panigrahi D, Rewatkar P, Haick H (2024) Role of machine learning assisted biosensors in point-of-care-testing for clinical decisions. ACS Sens 9:4495–4519. 10.1021/acssensors.4c01582 39145721 PMC11443532

[10] Deshmukh MT, Thakre Y, Bisen WH, Sankar K, Avthankar A, Shahade AK, Bhaiyya M, Kulkarni MB (2026) From drops to decisions: AI/ML-driven biofluidics for clinical diagnostics and healthcare intelligence. Anal Chem 98:24–42. 10.1021/acs.analchem.5c06356 41433142 PMC12809642

[11] Wankhede PR, Bhuyar D, Zanwar S, Pawar R, Jadhav MR, Gandhewar N, Kulkarni MB, Bhaiyya M, Haick H (2025) Artificial intelligence for noninvasive health diagnostics. ACS Sens 10:8217–8255. 10.1021/acssensors.5c03171 41165316

[12] Drain PK (2024) Point-of-care diagnostics (POCD) in resource-limited settings. Diagnostics. 10.3390/diagnostics14171926 39272710 PMC11394390

[13] Visseren FLJ, Mach F, Smulders YM, Carballo D, Koskinas KC, Bäck M, Benetos A, Biffi A, Boavida J-M, Capodanno D, Cosyns B, Crawford C, Davos CH, Desormais I, Di Angelantonio E, Franco OH, Halvorsen S, Hobbs FDR, Hollander M, Jankowska EA, Michal M, Sacco S, Sattar N, Tokgozoglu L, Tonstad S, Tsioufis KP, van Dis I, van Gelder IC, Wanner C, Williams B (2021) 2021 ESC guidelines on cardiovascular disease prevention in clinical practice. Eur Heart J 42:3227–3337. 10.1093/eurheartj/ehab484 34458905

[14] Perone F, Bernardi M, Redheuil A, Mafrica D, Conte E, Spadafora L, Ecarnot F, Tokgozoglu L, Santos-Gallego CG, Kaiser SE, Fogacci F, Sabouret A, Bhatt DL, Paneni F, Banach M, Santos R, Biondi Zoccai G, Ray KK, Sabouret P (2023) Role of cardiovascular imaging in risk assessment: recent advances, gaps in evidence, and future directions. J Clin Med. 10.3390/jcm12175563 37685628 PMC10487991

[15] Golub IS, Termeie OG, Kristo S, Schroeder LP, Lakshmanan S, Shafter AM, Hussein L, Verghese D, Aldana-Bitar J, Manubolu VS, Budoff MJ (2023) Major global coronary artery calcium guidelines. JACC Cardiovasc Imaging 16:98–117. 10.1016/j.jcmg.2022.06.018 36599573

[16] Nassanga R, Nakasujja N, Jones SE, Mubuuke AG, Sajatovic M, Lwere K, Kawooya MG, Ocan M, Kaddumukasa M (2025) Brain MRI for the diagnosis of dementia in low- and middle-income countries: a systematic review and meta-analysis protocol of diagnostic utility and imaging findings. BMJ Open 15:e108417. 10.1136/bmjopen-2025-108417 41248402 PMC12625966

[17] Andersen E, Casteigne B, Chapman WD, Creed A, Foster F, Lapins A, Shatz R, Sawyer RP (2021) Diagnostic biomarkers in Alzheimer’s disease. Biomark Neuropsychiatry 5:100041. 10.1016/j.bionps.2021.100041

[18] Dumas A, Destrebecq F, Esposito G, Suchonova D, Steen Frederiksen K (2023) Rethinking the detection and diagnosis of Alzheimer’s disease: outcomes of a European Brain Council project. Aging Brain 4:100093. 10.1016/j.nbas.2023.100093 37692977 PMC10483037

[19] Assudani PJ, Bhurgy AS, Kollem S, Bhurgy BS, Ahmad MO, Kulkarni MB, Bhaiyya M (2025) Artificial intelligence and machine learning in infectious disease diagnostics: a comprehensive review of applications, challenges, and future directions. Microchem J 218:115802. 10.1016/j.microc.2025.115802

[20] Zhu Z, Wang Y, Qi Z, Hu W, Zhang X, Wagner SK, Wang Y, Ran AR, Ong J, Waisberg E, Masalkhi M, Suh A, Tham YC, Cheung CY, Yang X, Yu H, Ge Z, Wang W, Sheng B, Liu Y, Lee AG, Denniston AK, van Wijngaarden P, Keane PA, Cheng C-Y, He M, Wong TY (2025) Oculomics: current concepts and evidence. Prog Retin Eye Res 106:101350. 10.1016/j.preteyeres.2025.101350 40049544

[21] Ghenciu LA, Dima M, Stoicescu ER, Iacob R, Boru C, Hațegan OA (2024) Retinal imaging-based oculomics: artificial intelligence as a tool in the diagnosis of cardiovascular and metabolic diseases. Biomedicines. 10.3390/biomedicines12092150 39335664 PMC11430496

[22] Bilal H, Keles A, Bendechache M (2025) Advances in disease detection through retinal imaging: a systematic review. Comput Biol Med 194:110412. 10.1016/j.compbiomed.2025.110412 40482560

[23] Untracht GR, Durkee MS, Zhao M, Kwok-Cheung Lam A, Sikorski BL, Sarunic MV, Andersen PE, Sampson DD, Chen FK, Sampson DM (2024) Towards standardising retinal OCT angiography image analysis with open-source toolbox OCTAVA. Sci Rep 14:5979. 10.1038/s41598-024-53501-6 38472220 PMC10933365

[24] Almidani L, Sabharwal J, Shahidzadeh A, Martinez AC, Ting SJ, Vaidya B, Jiang X, Kowalczyk T, Beiser A, Sobrin L, Seshadri S, Ramulu P, Kashani AH (2024) Biological and methodological variability in retinal nerve fiber layer OCT: the Framingham Heart Study. Ophthalmol Sci 4:100549. 10.1016/j.xops.2024.100549 39161752 PMC11331915

[25] Saeed A, Hadoux X, van Wijngaarden P (2025) Hyperspectral retinal imaging biomarkers of ocular and systemic diseases. Eye 39:667–672. 10.1038/s41433-024-03135-9 38778136 PMC11885810

[26] Williams DR, Burns SA, Miller DT, Roorda A (2023) Evolution of adaptive optics retinal imaging. Biomed Opt Express 14:1307–1338. 10.1364/BOE.485371 36950228 PMC10026580

[27] Tan YY, Kang HG, Lee CJ, Kim SS, Park S, Thakur S, Da Soh Z, Cho Y, Peng Q, Lee K, Tham Y-C, Rim TH, Cheng C (2024) Prognostic potentials of AI in ophthalmology: systemic disease forecasting via retinal imaging. Eye Vis 11:17. 10.1186/s40662-024-00384-3 PMC11071258 38711111

[28] Yang Q, Bee YM, Lim CC, Sabanayagam C, Yim-Lui Cheung C, Wong TY, Ting DSW, Lim L-L, Li H, He M, Lee AY, Shaw AJ, Keong YK, Wei Tan GS (2025) Use of artificial intelligence with retinal imaging in screening for diabetes-associated complications: systematic review. EClinicalMedicine 81:103089. 10.1016/j.eclinm.2025.103089 40052065 PMC11883405

[29] Zhou Y, Chia MA, Wagner SK, Ayhan MS, Williamson DJ, Struyven RR, Liu T, Xu M, Lozano MG, Woodward-Court P, Kihara Y, Altmann A, Lee AY, Topol EJ, Denniston AK, Alexander DC, Keane PA (2023) A foundation model for generalizable disease detection from retinal images. Nature 622:156–163. 10.1038/s41586-023-06555-x 37704728 PMC10550819

[30] El-Ateif S, Idri A (2024) Multimodality fusion strategies in eye disease diagnosis. J Imaging Inf Med 37:2524–2558. 10.1007/s10278-024-01105-x PMC11522204 38639808

[31] Liu Z, Hu Y, Qiu Z, Niu Y, Zhou D, Li X, Shen J, Jiang H, Li H, Liu J (2024) Cross-modal attention network for retinal disease classification based on multi-modal images. Biomed Opt Express 15:3699–3714. 10.1364/BOE.516764 38867787 PMC11166426

[32] Jin K, Yu T, Grzybowski A (2026) Multimodal artificial intelligence in ophthalmology: applications, challenges, and future directions. Surv Ophthalmol 71:158–167. 10.1016/j.survophthal.2025.07.003 40683606

[33] Chikumba S, Hu Y, Luo J (2023) Deep learning-based fundus image analysis for cardiovascular disease: a review. Ther Adv Chronic Dis 14:20406223231209896. 10.1177/20406223231209895 PMC10657535 38028950

[34] Li LY, Isaksen AA, Lebiecka-Johansen B, Funck K, Thambawita V, Byberg S, Andersen TH, Norgaard O, Hulman A (2024) Prediction of cardiovascular markers and diseases using retinal fundus images and deep learning: a systematic scoping review. Eur Heart J Digit Health 5:660–669. 10.1093/ehjdh/ztae068 39563905 PMC11570365

[35] Grzybowski A, Jin K, Zhou J, Pan X, Wang M, Ye J, Wong TY (2024) Retina fundus photograph-based artificial intelligence algorithms in medicine: a systematic review. Ophthalmol Ther 13:2125–2149. 10.1007/s40123-024-00981-4 38913289 PMC11246322

[36] Wang J, Wang YX, Zeng D, Zhu Z, Li D, Liu Y, Sheng B, Grzybowski A, Wong TY (2025) Artificial intelligence-enhanced retinal imaging as a biomarker for systemic diseases. Theranostics 15:3223–3233. 10.7150/thno.100786 40093903 PMC11905132

[37] Chua J, Tan B, Wong D, Garhöfer G, Liew XW, Popa-Cherecheanu A, Chin CL, Milea D, Chen CL-H, Schmetterer L (2024) Optical coherence tomography angiography of the retina and choroid in systemic diseases. Prog Retin Eye Res 103:101292. 10.1016/j.preteyeres.2024.101292 39218142

[38] Merriott DJ, Parikh D, Najac MJ, Duran LM, Haq A, Rosen RB, Chui TYP (2025) Optical coherence tomography and optical coherence tomography angiography in systemic disease. Taiwan J Ophthalmol 15 https://journals.lww.com/tjop/fulltext/2025/07000/optical_coherence_tomography_and_optical_coherence.5.aspx 10.4103/tjo.TJO-D-25-00053 PMC12456916 40995322

[39] Ganji Z, Nikparast F, Shoeibi N, Shoeibi A, Zare H, Sharak NA (2025) Retinal imaging and artificial intelligence: a systematic review and meta-analysis of diagnostic techniques for neurodegenerative diseases. Photodiagnosis Photodyn Ther 55:104788. 10.1016/j.pdpdt.2025.104788 40886956

[40] Lepoittevin M, Greig J, Erol O, Benani A, Bauvin P, Azzolini C, Donati S, Dubois B, Boscher C, Bodard S (2026) Retinal biomarkers for early Alzheimer’s detection: a systematic review of optical coherence tomography (OCT) findings. BMJ Open Ophthalmol. 10.1136/bmjophth-2025-002328 41565312 PMC12829385

[41] Kaštelan S, Gverović Antunica A, Puzović V, Didović Pavičić A, Čanović S, Kovačević P, Vučemilović PAF, Konjevoda S (2025) Non-invasive retinal biomarkers for early diagnosis of Alzheimer’s disease. Biomedicines. 10.3390/biomedicines13020283 40002697 PMC11852429

[42] Tang C, Zhang X, Miao H, Wang X, Liu J (2025) Retinal imaging techniques for neurodegenerative diseases: Parkinson’s disease and beyond. MedComm - Future Med 4:e70034. 10.1002/mef2.70034

[43] Alonso-Domínguez R, Vicente-García T, Arroyo-Romero S, Suárez-Moreno N, Navarro-Cáceres A, Domínguez-Martín A, Gómez-Sánchez L, Lugones-Sánchez C, García-Ortiz L, Ortega A, Gómez-Sánchez M, Navarro-Matias E, Gómez-Marcos MA (2025) Relationship between retinal vascular measurements and anthropometric indices in patients diagnosed with persistent COVID-19. J Clin Med. 10.3390/jcm14217857 41227253 PMC12610088

[44] Hanssen H, Streese L, Vilser W (2022) Retinal vessel diameters and function in cardiovascular risk and disease. Prog Retin Eye Res 91:101095. 10.1016/j.preteyeres.2022.101095 35760749

[45] Girach Z, Sarian A, Maldonado-García C, Ravikumar N, Sergouniotis PI, Rothwell PM, Frangi AF, Julian TH (2024) Retinal imaging for the assessment of stroke risk: a systematic review. J Neurol 271:2285–2297. 10.1007/s00415-023-12171-6 38430271 PMC11055692

[46] Theuerle JD, Al-Fiadh AH, Wong E, Patel SK, Ashraf G, Nguyen T, Wong TY, Ierino FL, Burrell LM, Farouque O (2022) Retinal microvascular function predicts chronic kidney disease in patients with cardiovascular risk factors. Atherosclerosis 341:63–70. 10.1016/j.atherosclerosis.2021.10.008 34756728

[47] London A, Benhar I, Schwartz M (2013) The retina as a window to the brain—from eye research to CNS disorders. Nat Rev Neurol 9:44–53. 10.1038/nrneurol.2012.227 23165340

[48] López-de-Eguileta A, López-García S, Lage C, Pozueta A, García-Martínez M, Kazimierczak M, Bravo M, Irure J, López-Hoyos M, Muñoz-Cacho P, Rodríguez-Perez N, Tordesillas-Gutiérrez D, Goikoetxea A, Nebot C, Rodríguez-Rodríguez E, Casado A, Sánchez-Juan P (2022) The retinal ganglion cell layer reflects neurodegenerative changes in cognitively unimpaired individuals. Alzheimers Res Ther 14:57. 10.1186/s13195-022-00998-6 35449033 PMC9022357

[49] Yuan A, Lee CS (2022) Retinal biomarkers for Alzheimer Disease: the facts and the future. Asia-Pac J Ophthalmol 11:140–148. 10.1097/APO.0000000000000505 PMC9889204 35533333

[50] Majeed A, Marwick B, Yu H, Fadavi H, Tavakoli M (2021) Ophthalmic biomarkers for Alzheimer’s disease: a review,. Front Aging Neurosci. 10.3389/fnagi.2021.720167 34566623 PMC8461312

[51] Deng Y, Jie C, Wang J, Liu Z, Li Y, Hou X (2022) Evaluation of retina and microvascular changes in the patient with Parkinson’s disease: a systematic review and meta-analysis,. Front Med. 10.3389/fmed.2022.957700 36186761 PMC9520292

[52] Barrett-Young A, Ambler A, Cheyne K, Guiney H, Kokaua J, Steptoe B, Tham YC, Wilson GA, Wong TY, Poulton R (2022) Associations between retinal nerve fiber layer and ganglion cell layer in middle age and cognition from childhood to adulthood. JAMA Ophthalmol 140:262–268. 10.1001/jamaophthalmol.2021.6082 35142821 PMC8832305

[53] Yue T, Shi Y, Luo S, Weng J, Wu Y, Zheng X (2022) The role of inflammation in immune system of diabetic retinopathy: molecular mechanisms, pathogenetic role and therapeutic implications. Front Immunol 13:1055087. 10.3389/fimmu.2022.1055087 36582230 PMC9792618

[54] Kang Q, Dai H, Jiang S, Yu L (2022) Advanced glycation end products in diabetic retinopathy and phytochemical therapy. Front Nutr 9:1037186. 10.3389/fnut.2022.1037186 36466410 PMC9716030

[55] Invernizzi A, Chhablani J, Viola F, Gabrielle PH, Zarranz-Ventura J, Staurenghi G (2023) Diabetic retinopathy in the pediatric population: pathophysiology, screening, current and future treatments. Pharmacol Res 188:106670. 10.1016/j.phrs.2023.106670 36681366

[56] Waheed NK, Rosen RB, Jia Y, Munk MR, Huang D, Fawzi A, Chong V, Nguyen QD, Sepah Y, Pearce E (2023) Optical coherence tomography angiography in diabetic retinopathy. Prog Retin Eye Res 97:101206. 10.1016/j.preteyeres.2023.101206 37499857 PMC11268430

[57] Rudraraju M, Narayanan SP, Somanath PR (2020) Regulation of blood-retinal barrier cell-junctions in diabetic retinopathy. Pharmacol Res 161:105115. 10.1016/j.phrs.2020.105115 32750417 PMC7755666

[58] Li H-Y, Yuan Y, Fu Y-H, Wang Y, Gao X-Y (2020) Hypoxia-inducible factor-1α: a promising therapeutic target for vasculopathy in diabetic retinopathy. Pharmacol Res 159:104924. 10.1016/j.phrs.2020.104924 32464323

[59] Abdollahi M, Jafarizadeh A, Ghafouri-Asbagh A, Sobhi N, Pourmoghtader K, Pedrammehr S, Asadi H, Tan R-S, Alizadehsani R, Acharya UR (2024) Artificial intelligence in assessing cardiovascular diseases and risk factors via retinal fundus images: a review of the last decade. WIREs Data Mining Knowl Discov 14:e1560. 10.1002/widm.1560

[60] Taufik SA, Ramli N, Tan AH, Lim S-Y, Ghani MTA, Shahrizaila N (2024) Longitudinal changes in the retinal nerve fiber layer thickness in Amyotrophic Lateral Sclerosis and Parkinson’s Disease. J Clin Neurol 20:285–292. 10.3988/jcn.2023.0353 38627230 PMC11076187

[61] Murueta-Goyena A, Romero-Bascones D, Teijeira-Portas S, Urcola JA, Ruiz-Martínez J, Del Pino R, Acera M, Petzold A, Wagner SK, Keane PA, Ayala U, Barrenechea M, Tijero B, Gómez Esteban JC, Gabilondo I (2024) Association of retinal neurodegeneration with the progression of cognitive decline in Parkinson’s disease. npj Park Dis 10:26. 10.1038/s41531-024-00637-x PMC10805713 38263165

[62] König M, Seeböck P, Gerendas BS, Mylonas G, Winklhofer R, Dimakopoulou I, Schmidt-Erfurth UM (2023) Quality assessment of colour fundus and fluorescein angiography images using deep learning. Br J Ophthalmol 108:98–104. 10.1136/bjo-2022-321963 36418144 PMC10804038

[63] Islam MM, Yang H-C, Poly TN, Jian W-S, Li Y-C(J (2020) Deep learning algorithms for detection of diabetic retinopathy in retinal fundus photographs: A systematic review and meta-analysis. Comput Methods Programs Biomed 191:105320. 10.1016/j.cmpb.2020.105320 32088490

[64] Dow ER, Khan NC, Chen KM, Mishra K, Perera C, Narala R, Basina M, Dang J, Kim M, Levine M, Phadke A, Tan M, Weng K, Do DV, Moshfeghi DM, Mahajan VB, Mruthyunjaya P, Leng T, Myung D (2023) AI-Human hybrid workflow enhances teleophthalmology for the detection of diabetic retinopathy. Ophthalmol Sci 3:100330. 10.1016/j.xops.2023.100330 37449051 PMC10336195

[65] Li Q, Li S, He Z, Guan H, Chen R, Xu Y, Wang T, Qi S, Mei J, Wang W (2020) DeepRetina: Layer segmentation of retina in OCT images using deep learning. Transl Vis Sci Technol 9:61. 10.1167/tvst.9.2.61 33329940 PMC7726589

[66] Zhou W-C, Tao J-X, Li J (2021) Optical coherence tomography measurements as potential imaging biomarkers for Parkinson’s disease: A systematic review and meta-analysis. Eur J Neurol 28:763–774. 10.1111/ene.14613 33107159

[67] Li D, Ran AR, Cheung CY, Prince JL (2023) Deep learning in optical coherence tomography: Where are the gaps? Clin Exp Ophthalmol 51:853–863. 10.1111/ceo.14258 37245525 PMC10825778

[68] Dhodapkar RM, Li E, Nwanyanwu K, Adelman R, Krishnaswamy S, Wang JC (2022) Deep learning for quality assessment of optical coherence tomography angiography images. Sci Rep 12:13775. 10.1038/s41598-022-17709-8 35962007 PMC9374672

[69] Abdelmotaal H, Sharaf M, Soliman W, Wasfi E, Kedwany SM (2022) Bridging the resources gap: Deep learning for fluorescein angiography and optical coherence tomography macular thickness map image translation. BMC Ophthalmol 22:355. 10.1186/s12886-022-02577-7 36050661 PMC9434904

[70] Parmar UPS, Arora A, Agarwal A, Gangaputra S, Agrawal R, Gupta V (2026) Detection and quantification of fluorescein angiography leakage: From manual grading to advances in machine learning. Surv Ophthalmol 71:613–626. 10.1016/j.survophthal.2025.08.015 40902824

[71] Tan T-E, Ibrahim F, Chandrasekaran PR, Teo KYC (2023) Clinical utility of ultra-widefield fluorescein angiography and optical coherence tomography angiography for retinal vein occlusions,. Front Med. 10.3389/fmed.2023.1110166 37359003 PMC10285461

[72] Dhirachaikulpanich D, Xie J, Chen X, Li X, Madhusudhan S, Zheng Y, Beare NAV (2024) Using deep learning to segment retinal vascular leakage and occlusion in retinal vasculitis. Ocul Immunol Inflamm 32:2291–2298. 10.1080/09273948.2024.2305185 38261457

[73] do Rio JM, Sen P, Rasheed R, Bagchi A, Nicholson L, Dubis AM, Bergeles C, Sivaprasad S (2020) Deep learning-based segmentation and quantification of retinal capillary non-perfusion on ultra-wide-field retinal fluorescein angiography,. J Clin Med. 10.3390/jcm9082537 32781564 PMC7464218

[74] Markowitz DM, Affel E, Hajnóczky G, Sergott RC (2025) Future applications of fluorescence lifetime imaging ophthalmoscopy in neuro-ophthalmology, neurology, and neurodegenerative conditions. Front Neurol 16:1493876. 10.3389/fneur.2025.1493876 40125394 PMC11927091

[75] Thiemann N, Sonntag SR, Kreikenbohm M, Böhmerle G, Stagge J, Grisanti S, Martinetz T, Miura Y (2024) Artificial intelligence in fluorescence lifetime imaging ophthalmoscopy (FLIO) data analysis-toward retinal metabolic diagnostics. Diagnostics Basel. 10.3390/diagnostics14040431 38396470 PMC10888399

[76] Zhang W, Tu X, Wang X, Lin D, Liu D, Lai W, Xu A, Wen J, Lin H (2025) Retinal oximetry: new insights into ocular and systemic diseases. Graefes Arch Clin Exp Ophthalmol 263:2101–2115. 10.1007/s00417-025-06831-8 40254630 PMC12414079

[77] Williams RC, Harrison WW, Carkeet A, Ostrin LA (2023) Twenty-four hour diurnal variation in retinal oxygen saturation. Vision Res 213:108314. 10.1016/j.visres.2023.108314 37657366 PMC11148934

[78] Jeong Y, Hong Y-J, Han J-H (2022) Review of machine learning applications using retinal fundus images. Diagnostics Basel. 10.3390/diagnostics12010134 35054301 PMC8774893

[79] Kankrale R, Kokare M (2025) Artificial intelligence in retinal image analysis for hypertensive retinopathy diagnosis: a comprehensive review and perspective. Vis Comput Ind Biomed Art 8:11. 10.1186/s42492-025-00194-x 40307650 PMC12044089

[80] Sonune BS, Udaykumar R, Nemane SG, Tulaskar DP, Bhaiyya M, Haick H (2026) Interpretable deep learning in dermoscopy: an XAI-driven ensemble for skin lesion diagnosis. IEEE Access 14:62126–62142. 10.1109/ACCESS.2026.3684257

[81] Marode TP, Bhangdiya VK, Nemane S, Tulaskar D, Sarad VM, Sankar K, Chopade S, Avthankar A, Bhaiyya M, Kulkarni MB (2026) Artificial intelligence meets nail diagnostics: emerging image-based sensing platforms for non-invasive disease detection. Bioengineering. 10.3390/bioengineering13010075 41596006 PMC12838109

[82] Zazueta LJG, Covarrubias BLL, Cota CXN, Briseño MV, Hipólito JIN, Rodríguez GJA (2025) Segmentation algorithms in fundus images: a review of digital image analysis techniques. Appl Sci Basel. 10.3390/app152111324

[83] Abdi AS, Abdulazeez AM (2025) A comprehensive review of deep learning in OCT image segmentation and classification. Med Novel Technol Devices 28:100396. 10.1016/j.medntd.2025.100396

[84] Wu J-H, Koseoglu ND, Jones C, Liu TYA (2023) Vision transformers: the next frontier for deep learning-based ophthalmic image analysis. Saudi J Ophthalmol Off J Saudi Ophthalmol Soc 37:173–178. 10.4103/sjopt.sjopt_91_23 PMC10701151 38074310

[85] Huang S-C, Pareek A, Jensen M, Lungren MP, Yeung S, Chaudhari AS (2023) Self-supervised learning for medical image classification: a systematic review and implementation guidelines. npj Digit Med 6:74. 10.1038/s41746-023-00811-0 37100953 PMC10131505

[86] Yellapragada B, Hornauer S, Snyder K, Yu S, Yiu G (2022) Self-supervised feature learning and phenotyping for assessing Age-Related Macular Degeneration using retinal fundus images. Ophthalmol Retina 6:116–129. 10.1016/j.oret.2021.06.010 34217854 PMC9482819

[87] Nguyen TD, Le D-T, Bum J, Kim S, Song SJ, Choo H (2023) Self-FI: self-supervised learning for disease diagnosis in fundus images. Bioengineering. 10.3390/bioengineering10091089 37760191 PMC10526021

[88] Engelmann J, Bernabeu MO (2025) Training a high-performance retinal foundation model with half-the-data and 400 times less compute. Nat Commun 16:6862. 10.1038/s41467-025-62123-z 40715051 PMC12297332

[89] Peng Y, Lin A, Wang M, Lin T, Liu L, Wu J, Zou K, Shi T, Feng L, Liang Z, Li T, Liang D, Yu S, Sun D, Luo J, Gao L, Chen X, Huang B, Zheng C, Jin C, Zheng D, Huang D, Li D, Zhang G, Wu H, Xia H, Lin H, Liang H, Yi J, Huang J, Liu J, Chen M, Zeng Q, Li T, Chen W, Huang X, Chen X, Ke X, Liao X, Wang Y, Huang Y, Cheng Y, Zhang Y, Xiong Y, Huang Y, Wu Z, Huang Z, Cheng C-Y, Fu H, Chen H (2025) Enhancing AI reliability: a foundation model with uncertainty estimation for optical coherence tomography-based retinal disease diagnosis. Cell Rep Med 6:101876. 10.1016/j.xcrm.2024.101876 39706192 PMC11866418

[90] Assudani PJ, P B, A AL, George G, Avthankar A, Tiwari A, Bhaiyya M, Kulkarni MB (2026) Biosensing technologies for foodborne pathogen detection and healthcare: principles, emerging materials, and intelligent platforms. Microchim Acta 193:231. 10.1007/s00604-026-07976-x PMC12971809 41803510

[91] Thakoor KA, Yao J, Bordbar D, Moussa O, Lin W, Sajda P, Chen RWS (2022) A multimodal deep learning system to distinguish late stages of AMD and to compare expert vs. AI ocular biomarkers. Sci Rep 12:2585. 10.1038/s41598-022-06273-w 35173191 PMC8850456

[92] Zedadra A, Salah-Salah MY, Zedadra O, Guerrieri A (2025) Multi-modal AI for multi-label retinal disease prediction using OCT and fundus images: a hybrid approach. Sensors Basel. 10.3390/s25144492 40732620 PMC12298238

[93] Morano J, Fazekas B, Sükei E, Fecso R, Emre T, Gumpinger M, Faustmann G, Oghbaie M, Schmidt-Erfurth U, Bogunović H (2025) Multimodal foundation model and benchmark for comprehensive retinal OCT image analysis. npj Digit Med 8:576. 10.1038/s41746-025-01852-3 40999048 PMC12462498

[94] Jin K, Yan Y, Chen M, Wang J, Pan X, Liu X, Liu M, Lou L, Wang Y, Ye J (2022) Multimodal deep learning with feature level fusion for identification of choroidal neovascularization activity in age-related macular degeneration. Acta Ophthalmol 100:e512–e520. 10.1111/aos.14928 34159761

[95] Yu Y, Zhu H (2023) Transformer-based cross-modal multi-contrast network for ophthalmic diseases diagnosis. Biocybern Biomed Eng 43:507–527. 10.1016/j.bbe.2023.06.001

[96] An S, Teo K, McConnell MV, Marshall J, Galloway C, Squirrell D (2025) AI explainability in oculomics: how it works, its role in establishing trust, and what still needs to be addressed. Prog Retin Eye Res 106:101352. 10.1016/j.preteyeres.2025.101352 40086660

[97] Patr\’\icio C, Neves JC, Teixeira LF (2023) Explainable deep learning methods in medical image classification: a survey. ACM Comput Surv. 10.1145/3625287

[98] Malerbi FK, Nakayama LF, Prado P, Yamanaka F, Melo GB, Regatieri CV, Stuchi JA (2024) Heatmap analysis for artificial intelligence explainability in diabetic retinopathy detection: illuminating the rationale of deep learning decisions. Ann Transl Med 12:89. 10.21037/atm-24-73 39507460 PMC11534741

[99] Bressler I, Dollberg D, Aviv R, Margalit D, Harris A, Siesky B, Ianchulev T, Dvey-Aharon Z (2025) Non-Contact Optical Blood Pressure Biometry Using AI-Based Analysis of Non-Mydriatic Fundus Imaging., MedRxiv Prepr. Serv Heal Sci. 10.1101/2025.01.06.25320084

[100] Poplin R, Varadarajan AV, Blumer K, Liu Y, McConnell MV, Corrado GS, Peng L, Webster DR (2018) Prediction of cardiovascular risk factors from retinal fundus photographs via deep learning. Nat Biomed Eng 2:158–164. 10.1038/s41551-018-0195-0 31015713

[101] Rim TH, Lee G, Kim Y, Tham Y-C, Lee CJ, Baik SJ, Kim YA, Yu M, Deshmukh M, Lee BK, Park S, Kim HC, Sabayanagam C, Ting DSW, Wang YX, Jonas JB, Kim SS, Wong TY, Cheng C-Y (2020) Prediction of systemic biomarkers from retinal photographs: development and validation of deep-learning algorithms. Lancet Digit Health 2:e526–e536. 10.1016/S2589-7500(20)30216-8 33328047

[102] Arnould L, Guenancia C, Bourredjem A, Binquet C, Gabrielle P-H, Eid P, Baudin F, Kawasaki R, Cottin Y, Creuzot-Garcher C, Jacquir S (2021) Prediction of cardiovascular parameters with supervised machine learning from Singapore “I” vessel assessment and OCT-Angiography: a pilot study. Transl Vis Sci Technol 10:20. 10.1167/tvst.10.13.20 34767626 PMC8590163

[103] White T, Selvarajah V, Wolfhagen-Sand F, Svangård N, Mohankumar G, Fenici P, Rough K, Onyango N, Lyons K, Mack C, Nduba V, Noorali Saleh M, Abayo I, Siddiqui A, Majdanska-Strzalka M, Kaszubska K, Hegelund-Myrback T, Esterline R, Manzur A, Parker VER (2024) Prediction of cardiovascular risk factors from retinal fundus photographs: Validation of a deep learning algorithm in a prospective non-interventional study in Kenya. Diabetes Obes Metab 26:2722–2731. 10.1111/dom.15587 38618987

[104] Squirrell DM, Yang S, Xie L, Ang S, Moghadam M, Vaghefi E, McConnell MV (2024) Blood pressure predicted from artificial intelligence analysis of retinal images correlates with future cardiovascular events. JACC Adv 3:101410. 10.1016/j.jacadv.2024.101410 39629061 PMC11612377

[105] Lee YC, Cha J, Shim I, Park W-Y, Kang SW, Lim DH, Won H-H (2023) Multimodal deep learning of fundus abnormalities and traditional risk factors for cardiovascular risk prediction. npj Digit Med 6:14. 10.1038/s41746-023-00748-4 36732671 PMC9894867

[106] Abbas Q, Daadaa Y, Rashid U, Sajid MZ, Ibrahim MEA (2023) HDR-EfficientNet: a classification of hypertensive and diabetic retinopathy using optimize EfficientNet architecture. Diagnostics. 10.3390/diagnostics13203236 37892058 PMC10606674

[107] Triwijoyo BK, Adil A, Zulfikri M (2025) Detection and classification of hypertensive retinopathy based on retinal image analysis using a deep learning approach. Comput Methods Programs Biomed 7:100191. 10.1016/j.cmpbup.2025.100191

[108] Kumar Y, Modi N, Koul A, Singh H (2026) Deep transfer learning and contour-based morphological analysis for detection of eye-hypertensive diseases from fundus images. Discov Artif Intell 6:53. 10.1007/s44163-026-00851-x

[109] Aghabeigi Alooghareh MM, Sheikhey MM, Sahafi A, Pirnejad H, Naemi A (2025) Deep learning for comprehensive analysis of retinal fundus images: detection of systemic and ocular conditions. Bioengineering. 10.3390/bioengineering12080840 40868353 PMC12383659

[110] Ding Y, Wei Z, Wang C, Li X, Li B, Liu X, Fu Z, Mo H, Zhang H (2025) Using deep learning to screen OCTA images for hypertension to reduce the risk of serious complications. Front Cell Dev Biol 13:1581785. 10.3389/fcell.2025.1581785 40703652 PMC12283777

[111] Coronado I, Abdelkhaleq R, Yan J, Marioni S.S., Jagolino-Cole A, Channa R, Pachade S, Sheth S.A., Giancardo L (2021) Towards Stroke Biomarkers on Fundus Retinal Imaging: A Comparison Between Vasculature Embeddings and General Purpose Convolutional Neural Networks. Annu Int Conf IEEE Eng Med Biol Soc IEEE Eng Med Biol Soc Annu Int Conf 2021:3873–3876. 10.1109/EMBC46164.2021.9629856 PMC8981508 34892078

[112] Zhu Z, Hu W, Chen R, Xiong R, Wang W, Shang X, Chen Y, Kiburg K, Shi D, He S, Huang Y, Zhang X, Tang S, Zeng J, Yu H, Yang X, He M (2022) Retinal age gap as a predictive biomarker of stroke risk. BMC Med 20:466. 10.1186/s12916-022-02620-w 36447293 PMC9710167

[113] Yusufu M, Friedman DS, Kang M, Padhye A, Shang X, Zhang L, Shi D, He M (2025) Retinal vascular fingerprints predict incident stroke: findings from the UK Biobank cohort study. Heart 111:306–313. 10.1136/heartjnl-2024-324705 39805634

[114] Jiang N, Ji H, Guan Z, Pan Y, Deng C, Guo Y, Liu D, Chen T, Wang S, Wu Y, Yang D, Ran AR, Hamzah H, Chee ML, Yin C, Thinggaard BS, Pedersen FN, Peng Q, Quek TC, Goh JHL, Singh S, Abd Raof AS, Lee-Boey JWS, Lu Y, Huang S, Shu J, Yu S, Jin Y, Li T, Qin Y, Wang J, Yang X, Hu T, Wang Z, Zhao Y, Lee S, Wei X, Zheng H, Li Y, Shen J, Zhou Y, Lin S, Wu C, Dai R, Ruan L, Hogg RE, Wright D, Wang YX, Zheng Y, Tan GSW, Sabanayagam C, Bao Y, Zhang C, Zhang P, Zou W, Guo M, Yang X, McKay GJ, Grauslund J, Lim L-L, Li Z, Cheung CY, Tham YC, Cheng C-Y, Wang Y, Dai Q, Jia W, Li H, Sheng B, Wong TY (2025) A deep learning system for detecting silent brain infarction and predicting stroke risk. Nat Biomed Eng 9:1907–1919. 10.1038/s41551-025-01413-9 40481238 PMC12623247

[115] Fu W, Zhou X, Wang M, Li P, Hou J, Gao P, Wang J (2022) Fundus changes evaluated by OCTA in patients with cerebral small vessel disease and their correlations: a cross-sectional study. Front Neurol. 10.3389/fneur.2022.843198 35547389 PMC9081972

[116] Geerling CF, Terheyden JH, Langner SM, Kindler C, Keil VC, Turski CA, Turski GN, Wintergerst MWM, Petzold GC, Finger RP (2022) Changes of the retinal and choroidal vasculature in cerebral small vessel disease. Sci Rep 12:3660. 10.1038/s41598-022-07638-x 35256658 PMC8901619

[117] Zhuo Y, Gao W, Wu Z, Jiang L, Luo Y, Ma X, Deng Z, Ma L, Wu J (2024) Evaluating retinal blood vessels for predicting white matter hyperintensities in ischemic stroke: A deep learning approach. J Stroke Cerebrovasc Dis 33:108070. 10.1016/j.jstrokecerebrovasdis.2024.108070 39393513

[118] Pachade S, Coronado I, Abdelkhaleq R, Yan J, Salazar-Marioni S, Jagolino A, Green C, Bahrainian M, Channa R, Sheth SA, Giancardo L (2022) Detection of stroke with retinal microvascular density and self-supervised learning using OCT-A and fundus imaging. J Clin Med. 10.3390/jcm11247408 36556024 PMC9788382

[119] Rim TH, Lee CJ, Tham Y-C, Cheung N, Yu M, Lee G, Kim Y, Ting DSW, Chong CCY, Choi YS, Yoo TK, Ryu IH, Baik SJ, Kim YA, Kim SK, Lee S-H, Lee BK, Kang S-M, Wong EYM, Kim HC, Kim SS, Park S, Cheng C-Y, Wong TY (2021) Deep-learning-based cardiovascular risk stratification using coronary artery calcium scores predicted from retinal photographs. Lancet Digit Health 3:e306–e316. 10.1016/S2589-7500(21)00043-1 33890578

[120] Barriada RG, Simó-Servat O, Planas A, Hernández C, Simó R, Masip D (2022) Deep learning of retinal imaging: a useful tool for coronary artery calcium score prediction in diabetic patients. Applied Sciences. 10.3390/app12031401

[121] Vaghefi E, Squirrell D, Yang S, An S, Xie L, Durbin MK, Hou H, Marshall J, Shreibati J, McConnell MV, Budoff M (2024) Development and validation of a deep-learning model to predict 10-year atherosclerotic cardiovascular disease risk from retinal images using the UK Biobank and EyePACS 10K datasets. Cardiovasc Digit Health J 5:59–69. 10.1016/j.cvdhj.2023.12.004 38765618 PMC11096659

[122] Qian Y, Li L, Nakashima Y, Nagahara H, Nishida K, Kawasaki R (2024) Is cardiovascular risk profiling from UK Biobank retinal images using explicit deep learning estimates of traditional risk factors equivalent to actual risk measurements? A prospective cohort study design. BMJ Open. 10.1136/bmjopen-2023-078609 39384229 PMC11474751

[123] Mellor J, Jiang W, Fleming A, McGurnaghan SJ, Blackbourn L, Styles C, Storkey AJ, McKeigue PM, Colhoun HM (2023) Can deep learning on retinal images augment known risk factors for cardiovascular disease prediction in diabetes? A prospective cohort study from the national screening programme in Scotland. Int J Med Inform 175:105072. 10.1016/j.ijmedinf.2023.105072 37167840

[124] Zhong P, Li Z, Lin Y, Peng Q, Huang M, Jiang L, Li C, Kuang Y, Cui S, Yu D, Yu H, Yang X (2022) Retinal microvasculature impairments in patients with coronary artery disease: an optical coherence tomography angiography study. Acta Ophthalmol 100:225–233. 10.1111/aos.14806 33629471

[125] Zong D, Xi H, Ni Y, Liang T, Li M, Zhou J, Liu H (2024) SS-OCTA assessment of fundus microvascular changes and their correlation with coronary lesion severity in severe coronary heart disease. Sci Rep 14:31931. 10.1038/s41598-024-83467-4 39738530 PMC11685621

[126] Gulshan V, Peng L, Coram M, Stumpe MC, Wu D, Narayanaswamy A, Venugopalan S, Widner K, Madams T, Cuadros J, Kim R, Raman R, Nelson PC, Mega JL, Webster DR (2016) Development and validation of a deep learning algorithm for detection of diabetic retinopathy in retinal fundus photographs. JAMA 316:2402–2410. 10.1001/jama.2016.17216 27898976

[127] Ting DSW, Cheung C-L, Lim G, Tan GSW, Quang ND, Gan A, Hamzah H, Garcia-Franco R, Yeo IS, Lee SY, Wong EYM, Sabanayagam C, Baskaran M, Ibrahim F, Tan NC, Finkelstein EA, Lamoureux EL, Wong IY, Bressler NM, Sivaprasad S, Varma R, Jonas JB, He MG, Cheng C-Y, Cheung GCM, Aung T, Hsu W, Lee ML, Wong TY (2017) Development and validation of a deep learning system for diabetic retinopathy and related eye diseases using retinal images from multiethnic populations with diabetes. JAMA 318:2211–2223. 10.1001/jama.2017.18152 29234807 PMC5820739

[128] Abràmoff MD, Lavin PT, Birch M, Shah N, Folk JC (2018) Pivotal trial of an autonomous AI-based diagnostic system for detection of diabetic retinopathy in primary care offices. npj Digit Med 1:39. 10.1038/s41746-018-0040-6 31304320 PMC6550188

[129] Gulshan V, Rajan RP, Widner K, Wu D, Wubbels P, Rhodes T, Whitehouse K, Coram M, Corrado G, Ramasamy K, Raman R, Peng L, Webster DR (2019) Performance of a deep-learning algorithm vs manual grading for detecting diabetic retinopathy in India. JAMA Ophthalmol 137:987–993. 10.1001/jamaophthalmol.2019.2004 31194246 PMC6567842

[130] Dai L, Wu L, Li H, Cai C, Wu Q, Kong H, Liu R, Wang X, Hou X, Liu Y, Long X, Wen Y, Lu L, Shen Y, Chen Y, Shen D, Yang X, Zou H, Sheng B, Jia W (2021) A deep learning system for detecting diabetic retinopathy across the disease spectrum. Nat Commun 12:3242. 10.1038/s41467-021-23458-5 34050158 PMC8163820

[131] Dai L, Sheng B, Chen T, Wu Q, Liu R, Cai C, Wu L, Yang D, Hamzah H, Liu Y, Wang X, Guan Z, Yu S, Li T, Tang Z, Ran A, Che H, Chen H, Zheng Y, Shu J, Huang S, Wu C, Lin S, Liu D, Li J, Wang Z, Meng Z, Shen J, Hou X, Deng C, Ruan L, Lu F, Chee M, Quek TC, Srinivasan R, Raman R, Sun X, Wang YX, Wu J, Jin H, Dai R, Shen D, Yang X, Guo M, Zhang C, Cheung CY, Tan GSW, Tham Y-C, Cheng C-Y, Li H, Wong TY, Jia W (2024) A deep learning system for predicting time to progression of diabetic retinopathy. Nat Med 30:584–594. 10.1038/s41591-023-02702-z 38177850 PMC10878973

[132] Betzler BK, Chee EYL, He F, Lim CC, Ho J, Hamzah H, Tan NC, Liew G, McKay GJ, Hogg RE, Young IS, Cheng C-Y, Lim SC, Lee AY, Wong TY, Lee ML, Hsu W, Tan GSW, Sabanayagam C (2023) Deep learning algorithms to detect diabetic kidney disease from retinal photographs in multiethnic populations with diabetes. J Am Med Inform Assoc 30:1904–1914. 10.1093/jamia/ocad179 37659103 PMC10654858

[133] Cervera DR, Smith L, Diaz-Santana L, Kumar M, Raman R, Sivaprasad S (2021) Identifying peripheral neuropathy in colour fundus photographs based on deep learning. Diagnostics. 10.3390/diagnostics11111943 34829290 PMC8623417

[134] Yun J-S, Kim J, Jung S-H, Cha S-A, Ko S-H, Ahn Y-B, Won H-H, Sohn K-A, Kim D (2022) A deep learning model for screening type 2 diabetes from retinal photographs. Nutr Metab Cardiovasc Dis 32:1218–1226. 10.1016/j.numecd.2022.01.010 35197214 PMC9018521

[135] Zhang K, Liu X, Xu J, Yuan J, Cai W, Chen T, Wang K, Gao Y, Nie S, Xu X, Qin X, Su Y, Xu W, Olvera A, Xue K, Li Z, Zhang M, Zeng X, Zhang CL, Li O, Zhang EE, Zhu J, Xu Y, Kermany D, Zhou K, Pan Y, Li S, Lai IF, Chi Y, Wang C, Pei M, Zang G, Zhang Q, Lau J, Lam D, Zou X, Wumaier A, Wang J, Shen Y, Hou FF, Zhang P, Xu T, Zhou Y, Wang G (2021) Deep-learning models for the detection and incidence prediction of chronic kidney disease and type 2 diabetes from retinal fundus images. Nat Biomed Eng 5:533–545. 10.1038/s41551-021-00745-6 34131321

[136] Sabanayagam C, Xu D, Ting DSW, Nusinovici S, Banu R, Hamzah H, Lim C, Tham Y-C, Cheung CY, Tai ES, Wang YX, Jonas JB, Cheng C-Y, Lee ML, Hsu W, Wong TY (2020) A deep learning algorithm to detect chronic kidney disease from retinal photographs in community-based populations. Lancet Digit Heal 2:e295–e302. 10.1016/S2589-7500(20)30063-7 33328123

[137] Kang E-C, Hsieh Y-T, Li C-H, Huang Y-J, Kuo C-F, Kang J-H, Chen K-J, Lai C-C, Wu W-C, Hwang Y-S (2020) Deep learning–based detection of early renal function impairment using retinal fundus images: model development and validation. JMIR Med Inform 8:e23472. 10.2196/23472 33139242 PMC7728538

[138] Meng Z, Guan Z, Yu S, Wu Y, Zhao Y, Shen J, Lim CC, Chen T, Yang D, Ran AR, He F, Hamzah H, Singh S, Abd Raof AS, Lee-Boey JWS, Lim S-K, Sun X, Ge S, Xu G, Su H, Cheng Y, Lu F, Liao X, Jin H, Deng C, Ruan L, Zhang C, Wu C, Dai R, Jin Y, Wang W, Li T, Liu R, Li J, Shu J, Lu Y, Wang X, Wu Q, Qin Y, Tang J, Sheng X, Jiao Q, Yang X, Guo M, McKay GJ, Hogg RE, Liew G, Chee EYL, Hsu W, Lee ML, Szeto S, Luk AOY, Chan JCN, Cheung CY, Tan GSW, Tham Y-C, Cheng C-Y, Sabanayagam C, Lim L-L, Jia W, Li H, Sheng B, Wong TY (2025) Non-invasive biopsy diagnosis of diabetic kidney disease via deep learning applied to retinal images: a population-based study. Lancet Digit Health. 10.1016/j.landig.2025.02.008 40312169

[139] Govindaiah A, Bhuiyan T, Smith RT, Dhamoon MS, Bhuiyan A (2025) A machine learning prediction model to identify individuals at risk of 5-year incident stroke based on retinal imaging. Sensors (Basel). 10.3390/s25061917 40293071 PMC11946667

[140] Tian J, Smith G, Guo H, Liu B, Pan Z, Wang Z, Xiong S, Fang R (2021) Modular machine learning for Alzheimer’s disease classification from retinal vasculature. Sci Rep 11:238. 10.1038/s41598-020-80312-2 33420208 PMC7794289

[141] Cheung CY, Ran AR, Wang S, Chan VTT, Sham K, Hilal S, Venketasubramanian N, Cheng C-Y, Sabanayagam C, Tham YC, Schmetterer L, McKay GJ, Williams MA, Wong A, Au LWC, Lu Z, Yam JC, Tham CC, Chen JJ, Dumitrascu OM, Heng P-A, Kwok TCY, Mok VCT, Milea D, Chen CL-H, Wong TY (2022) A deep learning model for detection of Alzheimer’s disease based on retinal photographs: a retrospective, multicentre case-control study. Lancet Digit Heal 4:e806–e815. 10.1016/S2589-7500(22)00169-8 36192349

[142] Dumitrascu OM, Li X, Zhu W, Woodruff BK, Nikolova S, Sobczak J, Youssef A, Saxena S, Andreev J, Caselli RJ, Chen JJ, Wang Y (2024) Color fundus photography and deep learning applications in Alzheimer disease. Mayo Clin Proc Digit Heal 2:548–558. 10.1016/j.mcpdig.2024.08.005 PMC11695061 39748801

[143] Corbin D, Lesage F (2022) Assessment of the predictive potential of cognitive scores from retinal images and retinal fundus metadata via deep learning using the CLSA database. Sci. Rep. 12:5767. 10.1038/s41598-022-09719-3 35388080 PMC8986784

[144] Shi XH, Ju L, Dong L, Zhang RH, Shao L, Yan YN, Wang YX, Fu XF, Chen YZ, Ge ZY, Wei WB (2024) Deep learning models for the screening of cognitive impairment using multimodal fundus images. Ophthalmology Retina 8:666–677. 10.1016/j.oret.2024.01.019 38280426

[145] Xie J, Yi Q, Wu Y, Zheng Y, Liu Y, Macerollo A, Fu H, Xu Y, Zhang J, Behera A, Fan C, Frangi AF, Liu J, Lu Q, Qi H, Zhao Y (2024) Deep segmentation of OCTA for evaluation and association of changes of retinal microvasculature with Alzheimer’s disease and mild cognitive impairment. Br J Ophthalmol 108:432–439. 10.1136/bjo-2022-321399 36596660 PMC10894818

[146] Hao J, Kwapong WR, Shen T, Fu H, Xu Y, Lu Q, Liu S, Zhang J, Liu Y, Zhao Y, Zheng Y, Frangi AF, Zhang S, Qi H, Zhao Y (2024) Early detection of dementia through retinal imaging and trustworthy AI. NPJ Digit Med 7:294. 10.1038/s41746-024-01292-5 39428420 PMC11491446

[147] Dallora AL, Alexander J, Palesetti PP, Guenot D, Selvander M, Berglund JS, Behrens A (2025) Hyperspectral retinal imaging to detect Alzheimer’s disease in a memory clinic setting. Alzheimers Res Ther 17:232. 10.1186/s13195-025-01887-4 41153055 PMC12570430

[148] Sim MA, Tham YC, Nusinovici S, Quek TC, Yu M, Xue CC, Chee ML, Peng QS, Tan ESJ, Chan SP, Cai Y, Chong EJY, Tan BY, Venketasubramanian N, Hilal S, Lai MKP, Choi H, Richards AM, Cheng C-Y, Chen CLH (2025) A deep-learning retinal aging biomarker for cognitive decline and incident dementia. Alzheimers Dement 21:e14601. 10.1002/alz.14601 40042460 PMC11881618

[149] Wagner SK, Romero-Bascones D, Cortina-Borja M, Williamson DJ, Struyven RR, Zhou Y, Patel S, Weil RS, Antoniades CA, Topol EJ, Korot E, Foster PJ, Balaskas K, Ayala U, Barrenechea M, Gabilondo I, Schapira AHV, Khawaja AP, Patel PJ, Rahi JS, Denniston AK, Petzold A, Keane PA (2023) Retinal optical coherence tomography features associated with incident and prevalent Parkinson disease. Neurology 101:e1581–e1593. 10.1212/WNL.0000000000207727 37604659 PMC10585674

[150] Tran C, Shen K, Liu K, Ashok A, Ramirez-Zamora A, Chen J, Li Y, Fang R (2024) Deep learning predicts prevalent and incident Parkinson’s disease from UK Biobank fundus imaging. Sci Rep 14:3637. 10.1038/s41598-024-54251-1 38351326 PMC10864361

[151] Ahn S, Shin J, Song SJ, Yoon WT, Sagong M, Jeong A, Kim JH, Yu HG (2023) Neurologic dysfunction assessment in Parkinson disease based on fundus photographs using deep learning. JAMA Ophthalmol 141:234–240. 10.1001/jamaophthalmol.2022.5928 36757713 PMC9912166

[152] Ganji Z, Nikparast F, Shoeibi N, Shoeibi A, Zare H (2025) Decoding Parkinson’s diagnosis: an OCT-based explainable AI with SHAP/LIME transparency from the Persian Cohort Study. Photodiagnosis Photodyn Ther 54:104668. 10.1016/j.pdpdt.2025.104668 40523575

[153] Hasanshahi M, Mehdizadeh A, Mahmoudi T, Ostovan VR, Nowroozzadeh MH, Parsaei H (2026) An ensemble machine learning classifier for Parkinson’s disease diagnosis using optical coherence tomography angiography. Sci Rep. 10.1038/s41598-026-38407-9 41639547 PMC12923507

[154] Hu W, Wang W, Wang Y, Chen Y, Shang X, Liao H, Huang Y, Bulloch G, Zhang S, Kiburg K, Zhang X, Tang S, Yu H, Yang X, He M, Zhu Z (2022) Retinal age gap as a predictive biomarker of future risk of Parkinson’s disease. Age Ageing. 10.1093/ageing/afac062 35352798 PMC8966015

[155] Richardson A, Kundu A, Henao R, Lee T, Scott BL, Grewal DS, Fekrat S (2024) Multimodal retinal imaging classification for Parkinson’s disease using a convolutional neural network. Transl Vis Sci Technol 13:23. 10.1167/tvst.13.8.23 39136960 PMC11323992

[156] Hu W, Li K, Gagnon J, Wang Y, Raney T, Chen J, Chen Y, Okunuki Y, Chen W, Zhang B (2025) FundusNet: a deep-learning approach for fast diagnosis of neurodegenerative and eye diseases using fundus images. Bioengineering. 10.3390/bioengineering12010057 39851331 PMC11762182

[157] He Y, Carass A, Liu Y, Jedynak BM, Solomon SD, Saidha S, Calabresi PA, Prince JL (2019) Deep learning based topology guaranteed surface and MME segmentation of multiple sclerosis subjects from retinal OCT. Biomed Opt Express 10:5042–5058. 10.1364/BOE.10.005042 31646029 PMC6788619

[158] He Y, Carass A, Liu Y, Calabresi PA, Saidha S, Prince JL (2023) Longitudinal deep network for consistent OCT layer segmentation. Biomed Opt Express 14:1874–1893. 10.1364/BOE.487518 37206119 PMC10191669

[159] Pérez Del Palomar A, Cegoñino J, Montolío A, Orduna E, Vilades E, Sebastián B, Pablo LE, Garcia-Martin E (2019) Swept source optical coherence tomography to early detect multiple sclerosis disease. The use of machine learning techniques. PLoS One 14:e0216410. 10.1371/journal.pone.0216410 31059539 PMC6502323

[160] Montolío A, Martín-Gallego A, Cegoñino J, Orduna E, Vilades E, Garcia-Martin E, del Palomar AP (2021) Machine learning in diagnosis and disability prediction of multiple sclerosis using optical coherence tomography. Comput Biol Med 133:104416. 10.1016/j.compbiomed.2021.104416 33946022

[161] Dongil-Moreno FJ, Ortiz M, Pueyo A, Boquete L, Sánchez-Morla EM, Jimeno-Huete D, Miguel JM, Barea R, Vilades E, Garcia-Martin E (2024) Diagnosis of multiple sclerosis using optical coherence tomography supported by explainable artificial intelligence. Eye (Lond) 38:1502–1508. 10.1038/s41433-024-02933-5 38297153 PMC11126721

[162] Hernandez M, Ramon-Julvez U, Vilades E, Cordon B, Mayordomo E, Garcia-Martin E (2023) Explainable artificial intelligence toward usable and trustworthy computer-aided diagnosis of multiple sclerosis from optical coherence tomography. PLoS One 18:1–32. 10.1371/journal.pone.0289495 PMC10406231 37549174

[163] Ortiz M, Mallen V, Boquete L, Sánchez-Morla EM, Cordón B, Vilades E, Dongil-Moreno FJ, Miguel-Jiménez JM, Garcia-Martin E (2023) Diagnosis of multiple sclerosis using optical coherence tomography supported by artificial intelligence. Mult Scler Relat Disord. 10.1016/j.msard.2023.104725 37086637

[164] Khodabandeh Z, Rabbani H, Ashtari F, Zimmermann HG, Motamedi S, Brandt AU, Paul F, Kafieh R (2023) Discrimination of multiple sclerosis using OCT images from two different centers. Mult Scler Relat Disord 77:104846. 10.1016/j.msard.2023.104846 37413855

[165] Motamedi S, Yadav SK, Kenney RC, Lin T-Y, Kauer-Bonin J, Zimmermann HG, Galetta SL, Balcer LJ, Paul F, Brandt AU (2022) Prior optic neuritis detection on peripapillary ring scans using deep learning. Ann Clin Transl Neurol 9:1682–1691. 10.1002/acn3.51632 36285339 PMC9639624

[166] Rogaczewska M, Michalak S, Stopa M (2021) Differentiation between multiple sclerosis and neuromyelitis optica spectrum disorder using optical coherence tomography angiography. Sci Rep 11:10697. 10.1038/s41598-021-90036-6 34021191 PMC8140093

[167] Ciftci Kavaklioglu B, Erdman L, Goldenberg A, Kavaklioglu C, Alexander C, Oppermann HM, Patel A, Hossain S, Berenbaum T, Yau O, Yea C, Ly M, Costello F, Mah JK, Reginald A, Banwell B, Longoni G (2022) Ann Yeh, Machine learning classification of multiple sclerosis in children using optical coherence tomography. Mult Scler 28:2253–2262. 10.1177/13524585221112605 35946086 PMC9679797

[168] Nusinovici S, Rim TH, Yu M, Lee G, Tham Y-C, Cheung N, Chong CCY, Soh ZD, Thakur S, Lee CJ, Sabanayagam C, Lee BK, Park S, Kim SS, Kim HC, Wong T-Y, Cheng C-Y (2022) Retinal photograph-based deep learning predicts biological age, and stratifies morbidity and mortality risk. Age Ageing. 10.1093/ageing/afac065 35363255 PMC8973000

[169] Zhu Z, Shi D, Guankai P, Tan Z, Shang X, Hu W, Liao H, Zhang X, Huang Y, Yu H, Meng W, Wang W, Ge Z, Yang X, He M (2023) Retinal age gap as a predictive biomarker for mortality risk. Br J Ophthalmol 107:547–554. 10.1136/bjophthalmol-2021-319807 35042683

[170] Welikala RA, Fajtl J, Johnson G, Rahman F, Podoleanu R, Remagnino P, Freeman EE, Chambers R, Bolter L, Anderson J, Olvera-Barrios A, Warwick A, Foster PJ, Shakespeare R, Ganguly R, Egan C, Tufail A, Owen CG, Rudnicka AR, Barman SA, Datasets NELDESP (2024) 2024 IEEE EMBS Int Conf Biomed Heal Inf 1–8. 10.1109/BHI62660.2024.10913687

[171] Shakespeare R, Rudnicka AR, Welikala R, Barman SA, Khawaja AP, Foster PJ, Diagnosis (2025) Assess \& Dis Monit 17:e270087. 10.1002/dad2.70087 PMC11848343 39996031

[172] Cho B-J, Lee M, Han J, Kwon S, Oh MS, Yu K-H, Lee B-C, Kim JH, Kim C (2022) Prediction of white matter hyperintensity in brain MRI using fundus photographs via deep learning. J Clin Med. 10.3390/jcm11123309 35743380 PMC9224833

[173] Wisely CE, Richardson A, Henao R, Robbins CB, Ma JP, Wang D, Johnson KG, Liu AJ, Grewal DS, Fekrat S (2024) A Convolutional Neural Network Using Multimodal Retinal Imaging for Differentiation of Mild Cognitive Impairment from Normal Cognition. Ophthalmol Sci 4:100355. 10.1016/j.xops.2023.100355 37877003 PMC10591009

[174] Lim JI, Rachitskaya AV, Hallak JA, Gholami S, Alam MN (2024) Artificial intelligence for retinal diseases. Asia-Pacific J Ophthalmol 13:100096. 10.1016/j.apjo.2024.100096 39209215

[175] Ran J, Zhang G, Xia F, Zhang X, Xie J, Zhang H (2024) Source-free active domain adaptation for diabetic retinopathy grading based on ultra-wide-field fundus images. Comput Biol Med 174:108418. 10.1016/j.compbiomed.2024.108418 38593641

[176] Wei Y, Deng Y, Sun C, Lin M, Jiang H, Peng Y (2024) Deep learning with noisy labels in medical prediction problems: A scoping review. J Am Med Inf Assoc 31:1596–1607. 10.1093/jamia/ocae108 PMC11187424 38814164

[177] Shi J, Zhang K, Guo C, Yang Y, Xu Y, Wu J (2024) A survey of label-noise deep learning for medical image analysis, Med. Image Anal 95:103166. 10.1016/j.media.2024.103166 38613918

[178] Jan CL, Joseph S, Vingrys AJ, Henwood J, Ge Z, Stafford RS, He M (2025) Prospective pragmatic trial of automated retinal photography and AI glaucoma screening in Australian primary care. npj Digit Med 8:386. 10.1038/s41746-025-01768-y 40595445 PMC12216711

[179] Wolf RM, Channa R, Liu TYA, Zehra A, Bromberger L, Patel D, Ananthakrishnan A, Brown EA, Prichett L, Lehmann HP, Abramoff MD (2024) Autonomous artificial intelligence increases screening and follow-up for diabetic retinopathy in youth: The ACCESS randomized control trial. Nat Commun 15:421. 10.1038/s41467-023-44676-z 38212308 PMC10784572

[180] Williamson DJ, Struyven RR, Antaki F, Chia MA, Wagner SK, Jhingan M, Wu Z, Guymer R, Skene SS, Tammuz N, Thomson B, Chopra R, Keane PA (2024) Artificial intelligence to facilitate clinical trial recruitment in age-related macular degeneration. Ophthalmology Science. 10.1016/j.xops.2024.100566 39139546 PMC11321286

[181] Wang S, He X, Jian Z, Li J, Xu C, Chen Y, Liu Y, Chen H, Huang C, Hu J, Liu Z (2024) Advances and prospects of multi-modal ophthalmic artificial intelligence based on deep learning: A review. Eye Vis 11:38. 10.1186/s40662-024-00405-1 PMC11443922 39350240

[182] Wang M, Fan S, Li Y, Xie Z, Chen H (2025) Missing-modality enabled multi-modal fusion architecture for medical data. J Biomed Inform 164:104796. 10.1016/j.jbi.2025.104796 39988001

[183] Zou K, Chen Z, Yuan X, Shen X, Wang M, Fu H (2023) A review of uncertainty estimation and its application in medical imaging. Meta-Radiol 1:100003. 10.1016/j.metrad.2023.100003

[184] Dawood T, Chen C, Sidhu BS, Ruijsink B, Gould J, Porter B, Elliott MK, Mehta V, Rinaldi CA, Puyol-Antón E, Razavi R, King AP (2023) Uncertainty aware training to improve deep learning model calibration for classification of cardiac MR images. Med Image Anal 88:102861. 10.1016/j.media.2023.102861 37327613 PMC10788705

[185] Chen H, Gomez C, Huang C-M, Unberath M (2022) Explainable medical imaging AI needs human-centered design: Guidelines and evidence from a systematic review. npj Digit Med 5:156. 10.1038/s41746-022-00699-2 36261476 PMC9581990

[186] Zhang J, Chao H, Dasegowda G, Wang G, Kalra MK, Yan P (2024) Revisiting the trustworthiness of saliency methods in radiology AI. Radiol Artif Intell 6:e220221. 10.1148/ryai.220221 38166328 PMC10831523

[187] Xu J, Glicksberg BS, Su C, Walker P, Bian J, Wang F (2021) Federated learning for healthcare informatics. J Healthc Inf Res 5:1–19. 10.1007/s41666-020-00082-4 PMC7659898 33204939

[188] Raza A, Guzzo A, Ianni M, Lappano R, Zanolini A, Maggiolini M, Fortino G (2025) Federated learning in radiomics: a comprehensive meta-survey on medical image analysis. Comput Methods Programs Biomed 267:108768. 10.1016/j.cmpb.2025.108768 40279838

[189] Kamel Rahimi A, Pienaar O, Ghadimi M, Canfell OJ, Pole JD, Shrapnel S, van der Vegt AH, Sullivan C (2024) Implementing AI in hospitals to achieve a learning health system: systematic review of current enablers and barriers. J Med Internet Res 26:e49655. 10.2196/49655 39094106 PMC11329852

[190] Tun HM, Rahman HA, Naing L, Malik OA (2025) Trust in artificial intelligence–based clinical decision support systems among health care workers: systematic review. J Med Internet Res. 10.2196/69678 40772775 PMC12440830

[191] Park WY, Sippel Schmidt T, Salvador G, O’Donnell K, Genereaux B, Jeon K, You SC, Dewey BE, Nagy P (2025) Breaking data silos: incorporating the DICOM imaging standard into the OMOP CDM to enable multimodal research. J Am Med Inf Assoc 32:1533–1541. 10.1093/jamia/ocaf091 PMC12451937 40680297

[192] Iancu A, Bauer J, May MS, Prokosch H-U, Dörfler A, Uder M, Kapsner LA (2024) Large-scale integration of DICOM metadata into HL7-FHIR for medical research. Methods Inf Med 63:77–84. 10.1055/a-2521-4250 40233823 PMC12133321

[193] Wu W-T, Chao Y-W, Lin T-K, Huang C-K, Hsieh P-H (2025) Economic evaluation of AI-assisted technologies in healthcare: a systematic review. J Food Drug Anal 33:487–500. 10.38212/2224-6614.3570 41525195 PMC12795340

[194] Akune Y, Kawasaki R, Goto R, Tamura H, Hiratsuka Y, Yamada M (2025) Cost-effectiveness of AI-based diabetic retinopathy screening in nationwide health checkups and diabetes management in Japan: a modeling study. Diabetes Res Clin Pract 221:112015. 10.1016/j.diabres.2025.112015 39892817

[195] Spaide RF, Fujimoto JG, Waheed NK (2015) Image artifacts in optical coherence tomography angiography. Retina 35:2163–2180. 10.1097/IAE.0000000000000765 26428607 PMC4712934

[196] Mansberger SL, Menda SA, Fortune BA, Gardiner SK, Demirel S (2017) Automated segmentation errors when using optical coherence tomography to measure retinal nerve fiber layer thickness in glaucoma. Am J Ophthalmol 174:1–8. 10.1016/j.ajo.2016.10.020 27818206 PMC5548380

[197] Akiyama K, Saito H, Aoki S, Shirato S, Iwase A, Sugimoto K, Sakata R, Honjo M, Aihara M (2024) Effect of magnification error and axial length on circumpapillary capillary density and retinal nerve fiber layer thickness. Sci Rep 14:18874. 10.1038/s41598-024-69864-9 39143152 PMC11324904

